# Plasma-based early screening and monitoring of *EGFR* mutations in NSCLC patients by a 3-color digital PCR assay

**DOI:** 10.1038/s41416-020-1024-2

**Published:** 2020-08-12

**Authors:** Xiang Song, Jian Gong, Xiaoling Zhang, Xiaoyan Feng, Hui Huang, Min Gao, Li Chu

**Affiliations:** 1 Department of Thoracic Surgery, Cangzhou Central Hospital, 061000 Hebei, China; 2 Hebei Medical University, 050017 Hebei, China; 3 Department of Pathology, Cangzhou Central Hospital, 061000 Hebei, China; 4 Department of Research and Development, Apexbio Biotech (Suzhou) Co., Ltd., 215004 Suzhou, China; 5 School of Pharmacy, Hebei University of Chinese Medicine, 050200 Hebei, China; 6 Hebei Key Laboratory of integrative Medicine on Liver-Kidney Patterns, 050200 Hebei, China

**Keywords:** Pathology, Targeted therapies, Cancer screening, Diagnostics

## Abstract

**Background:**

Noninvasive plasma-based detection of *EGFR* mutations using digital PCR promises a fast, sensitive and reliable approach to predicting the efficiency of EGFR-TKI. However, the low throughput and high cost of digital PCR restricts its clinical application.

**Methods:**

We designed a digital PCR assay, which can simultaneously detect 39 mutations of exons 18–21 of the *EGFR* gene. To assess overall performance, retrospective FFPE tissues from 30 NSCLC patients and plasma from 33 NSCLC patients were collected and analysed.

**Results:**

The LoD of the *EGFR* mutations was as low as 0.308 copies/μL, and the linear correlation between the detected and expected values at different concentrations (0.01–10%) was low as well. Compared to ARMS-PCR in FFPE, the accuracy values of the dEGFR39 assay in plasma from 33 patients was 87.88% (29/33, 95% CI 72.67–95.18%). While monitoring the 33 patients, the *EGFR* mutation load as assessed by dEGFR39 was associated with the objective response to treatment. Thirteen samples from eight patients were identified by dEGFR39 to harbour the T790M mutation over time; of these patients, only nine (69%) were detected using SuperARMS.

**Conclusion:**

Our results indicate that dEGFR39 assay is reliable, sensitive and cost-efficient. This method is beneficial for profiling *EGFR* mutations for precision therapy and prognosis after TKI treatment, especially in patients with insufficient tissue biopsy samples.

## Background

In patients with non-small-cell lung cancer (NSCLC), the possibility of treatment with tyrosine kinase inhibitor (TKI) is determined by the presence of mutations on exons 18–21 of the epidermal growth factor receptor (*EGFR*).^1^ Clinical evidence heightens the fact that EGFR-targeted therapy can significantly improve progression-free survival (PFS) and overall survival (OS) in patients.^2,3^ Therefore, assessing the status of *EGFR* mutations is important for potential EGFR-TKI therapy.

Previous methods of *EGFR* mutation detection have been based on invasive approaches, such as surgical resection of tumours and needle biopsies.^4^ However, the heterogeneity of tumour tissue usually confounds the analysis of *EGFR* mutation load.^5^ Interestingly, recent data have shown that *EGFR* mutations can be found in plasma-derived cell-free DNA (cfDNA) from NSCLC patients. Moreover, studies have confirmed that *EGFR* mutations from plasma can predict the clinical response to targeted therapy.^6,7^

Based on plasma-derived DNA, noninvasive detection approaches have been developed, such as super amplification refractory mutation system (superARMS) PCR, next-generation sequencing (NGS), and digital PCR.^8–10^ Although ARMS-PCR is cost-efficient, its overall performance appears to be least sensitive. The NGS method, on the other hand, is more expensive. The digital PCR method features a quick turn-around and improved sensitivity, and is ideally used for the detection of known mutation types in cancer patients, especially in those whose tissue biopsy samples are insufficient.^11–14^ Digital PCR is a new approach to nucleic acid detection. The PCR reaction is first pressurised to partition into 30,000 droplets, each of which contains zero, one or more copies of the target molecule, and then PCR analysis is carried out. During amplification, TaqMan chemistry with dye-labelled probes is used to detect sequence-specific targets. The droplets containing the target molecule will generate fluorescence signals, which are defined as positive, while others are negative. According to the Poisson distribution, the fraction of negative reactions is used to generate an absolute count of the number of target molecules in the sample. Despite the fact that studies have shown good consistency of *EGFR* detection between plasma and tissue using digital PCR,^12,15,16^ the low throughput and high cost of digital PCR restricts its clinical application. There is not yet a method that simultaneously assesses all the driver mutations of the *EGFR* gene in plasma using digital PCR.^17,18^

Therefore, we developed an integrated digital PCR assay, named dEGFR39, which can unambiguously distinguish multiple mutations using three different fluorescence channels. This method is based on the need for low cost and small sample input. In this report, we provide evidence that dEGFR39 has excellent performance, which is not only useful to screen *EGFR-*targeted mutations from plasma of NSCLC patients to guide targeted therapy, but also to evaluate prognosis after treatment. To the best of our knowledge, this is the first report regarding the introduction of digital PCR for the screening and monitoring of all *EGFR* mutations from patient plasma.

## Methods

### Sample collection and processing

**Fig. 1:**
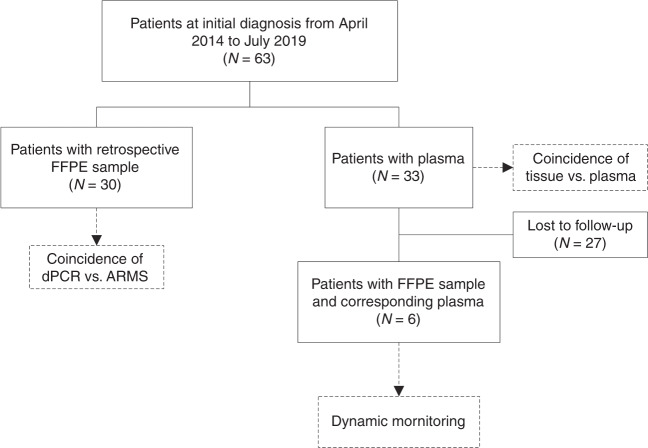
The scheme of the clinical study design.

The plasmid DNA of *EGFR* L858R, 19Del and T790M were prepared using the Apexbio plasmid DNA extraction kit, according to the manufacturer’s instructions (Apexbio, Suzhou, China). The human genomic DNA standard HD802 was obtained from Horizon (Horizon, Cambridge, UK). This study was approved by the ethics committee of Cangzhou Central Hospital, Hebei, China and all patients provided written informed consent. Patients were diagnosed with NSCLC and ARMS-PCR was performed to assess the mutation status of *EGFR*. For patient-derived FFPE tissues, we collectively obtained 63 samples, including 30 retrospective samples. Thirty-three patients were enrolled from May 2014 to June 2019. Plasma samples and CT images were collected from the participants every 2 months. The scheme of the clinical study design is shown in Fig. 1. Patient-derived FFPE tissues were cut into three pieces of 10-μm thick paraffin. FFPE DNA was extracted according to the procedure of the Qiagen FFPE DNA kit. Ten millilitre of peripheral blood was collected into a BCT DNA tube (Streck, La Vista, USA) and mixed by gentle inversion 10 times immediately. To separate plasma, we centrifuged the whole blood sample at 1600 × *g* for 10 min to obtain the supernatant, then centrifuged the supernatant at 16,000 × *g* for 10 min according to manufacturer’s instructions. Two millilitre of plasma was used to isolate DNA using the Qiagen circulating nucleic acid kit following the manufacturer’s instructions (Qiagen, Hilden, Germany). DNA quantification was obtained by the Colibri microvolume spectrophotometer (Titertek-Berthold, Pforzheim, Germany) and Qubit Fluorometer 2.0 (Invitrogen, California, USA).

### Detection of *EGFR* mutations using dEGFR39 assay

Oligonucleotides were synthesised by Sangon Biotech (Shanghai, China). Detection of the *EGFR* mutation using dEGFR39 assay (Apexbio, Suzhou, China) was carried out in three tubes on the Naica digital PCR system (Stilla Technologies, Villejuif, France) with Sapphire chips (Stilla Technologies, Villejuif, France). The list of 39 mutations found by the dEGFR39 assay is provided in Supplementary Table S1. Twenty-five microlitre of reaction mix in each tube contained 1X PerFecTa Multiplex qPCR ToughMix, 40 nM FITC (Saint Louis, MO, USA), 1 μl of a multiplex mix of primers and probes and 3 μl of DNA. The chip was loaded into the Naica Geode thermocycler to compartmentalise the droplets and to perform the PCR reaction. PCR consisted of 10 min at 95 °C, followed by 45 cycles of 95 °C for 20 s and 60 °C for 30 s. After amplification, the Sapphire chips were imaged using the Naica Prism3 reader.

### Limit of blank and limit of detection

The specificity and accuracy of dEGFR39 assay were assessed by wild-type (WT) and mutant DNA of *EGFR*. The limit of blank (LoB) and limit of detection (LoD) were determined as previously reported.^19^ To determine LoB, twenty replicates of a blank sample within two independent runs were carried out. The LoB was set as the highest mutant concentration that might be found when replicates of a blank sample are tested. The LoD was set as the lowest concentration that could be distinguished from the LoB with 95% certainty.^20^ The LoB and LoD were calculated with the following formulas:

1 $${\mathrm{LoB}} = {\mathrm{Mean}}_{{\mathrm{blank}}} + 1.645 \times {\mathrm{SD}}_{{\mathrm{blank}}}$$

2 $${\mathrm{LoD}} = {\mathrm{LoB}} + 1.645 \times {\mathrm{SD}}_{{\mathrm{low}}\,{\mathrm{concentration}}\,{\mathrm{sample}}}$$

Accuracy was assessed by testing the DNA Reference Standards and calculating the coefficient of variation and statistical differences.

### Data analysis

Raw data from digital PCR were analysed by Crystal Miner software (Stilla Technologies, Villejuif, France) according to the principle of Poisson distribution. In general, when there are λ targets per droplets, the fraction of positive droplets (P) is:

3 $${\it{P}} = 1 - {\it{e}}^{ - \lambda }$$

For each analysis involving data from two channels, where λ_c1_ and λ_c2_ are the average number of loading molecules in these two channels, with the total number of droplets N, the number of double-positive droplets in multiple digital PCR is:

4 $${\it{N}}_{{\mathrm{Dual}}} = {\it{N}} \times \left( {1 - {\it{e}}^{ - \lambda _{{\boldsymbol{c}}1}}} \right) \times \left( {1 - {\it{e}}^{ - \lambda _{{\boldsymbol{c}}2}}} \right)$$

NTC and *EGFR* Gene-Specific Multiplex Reference Standard genomic DNA HD802 were used as negative and positive controls, respectively. Negative and positive droplets were also used to check the fluorescence spill-over compensation. The paired *t*-test, Bland–Altman, and McNemar’s test were used to compare the consistency of different groups with PPA (sensitivity), NPA (specificity), accuracy (OPA) and Kappa values. The cut-off for statistical significance was *p* < 0.05. All statistical data were analysed by IBM SPSS statistics software 22.0 (IBM, Armonk, NY, USA) and GraphPad Prism 5 (GraphPad, La Jolla, CA, USA).

## Results

### Characteristics of dEGFR39 assay

In this study, dEGFR39 assay was developed to detect up to 39 mutations along exons 18–21 of the *EGFR* gene, including common mutation sites such as L858R, 19Del and T790M, as well as rare mutation sites such as L861Q, S768I, G719X, C797S and 20ins.

**Fig. 2:**
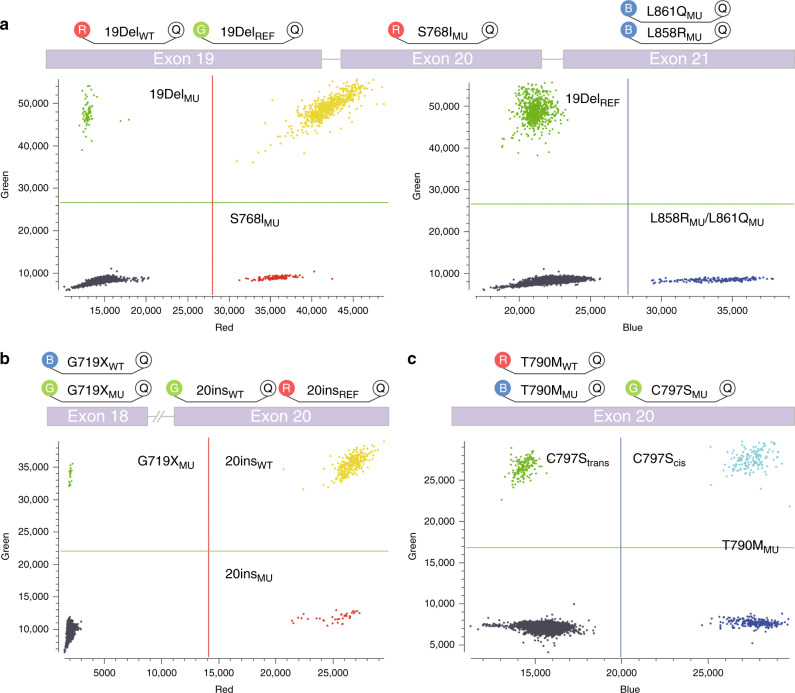
Illustration of dEGFR39 distribution in the three reactions and output data from digital PCR in the form of a 2D histogram. **a** 19DelWT molecules are double positive (CY5+/HEX+), and the rest of the signals in the HEX and CY5 channels are generated by 19DelMU (CY5−/HEX+) and S768IMU (CY5+/HEX−), respectively. The signals in the FAM channel are generated by L858RMU. **b** 20insWT molecules are double positive (CY5+/HEX+), and the rest of the signals in the HEX and CY5 channels are generated by G719XMU (CY5−/HEX+) and 20insMU (CY5+/HEX−), respectively. **c** The blue and green dots represent the signal of T790M and C797S in trans configuration with T790M, respectively. The double positive represents C797S in cis configuration with T790M.

To develop dEGFR39 assay, we designed digital PCR into three consecutive reactions. In the first reaction, 19Del_REF_ was labelled with HEX, L858R_MU_ and L861Q_MU_ were labelled with FAM, and 19Del_WT_ and S768I_MU_ were labelled with CY5 (Fig. 2). In the presence of 19Del, probe 19Del_WT_ could cross-react with the neighbouring nondeleted WT sequences; in the meantime, probe 19Del_REF_ was used to detect the *EGFR* gene (including 19Del_WT_ and 19Del_MU_). As a result, 19Del_WT_ molecules were double-positive (CY5+/HEX+), whereas the rest of the signals in the HEX and CY5 channels were only from 19Del_MU_ (CY5−/HEX+) and S768I_MU_ (CY5+/HEX−), respectively. The signals in the FAM channel were generated by L858R_MU_ and L861Q_MU_. Similarly, in the second reaction, G719X_WT_ and 20ins_REF_ were labelled with FAM and CY5, respectively, while 20ins_WT_ and G719X_MU_ were labelled with HEX. 20ins_WT_ molecules were consequently double-positive (CY5+/HEX+), and the rest of the signals in the HEX and CY5 channels were only from G719X_MU_ (CY5−/HEX+) and 20ins_MU_ (CY5+/HEX−), respectively. Given that the presence of C797S and T790M, whether in the trans or cis form, would determine the efficacy of TKI treatment,^21^ the third reaction was specifically designed to characterise this genotype. T790M_MU_ and C797S_MU_ were labelled with FAM and HEX, respectively. Since the amount of plasma-derived DNA was exceedingly low, the positive signal from the HEX channel could only be from C797S_MU_ in the trans configuration with T790M_MU_.

Notably, our assay included the C797S site, a critical mutation recently identified after TKI treatment.^22^ We did not detect C797S in patients enrolled in our study, which is consistent with previous reports that C797S does not occur in TKI-naïve NSCLC.^23^

### The specificity and sensitivity of dEGFR39

**Fig. 3:**
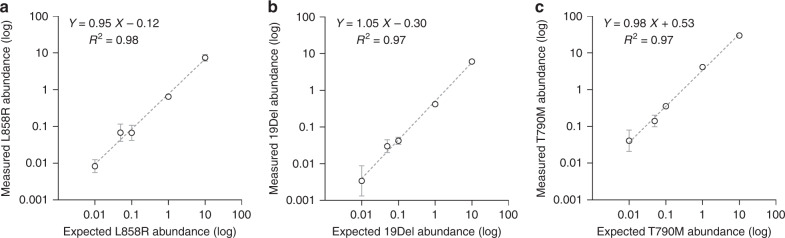
The linearity of EGFR L858R, 19Del, and T790M in the dEGFR39 assay. EGFR mutations were detected on a series of DNA with the mutant ratio of 10%, 1%, 0.1%, 0.05%, and 0.01%. Regression plot for the dilutions shows linearity and a good correlation for expected and measured values.

To assess the specificity of the dEGFR39 assay, both WT and mutant (MU) DNA of *EGFR* were synthesised. Notably, only a specific fluorescent signal could be detected that corresponded to WT and mutant forms (Supplementary Figs. S1–3), indicating the great specificity of the assay. To evaluate the accuracy, we performed the dEGFR39 reaction with a standard DNA HD802 (expected mutant ratio 12.5%). Our data showed the mutation loads of 19Del, T790M and L858R as being 12.63%, 12.19% and 12.13%, respectively. This assessment was in concordance with the expected values (*p* > 0.05) (Table 1), strongly suggesting that dEGFR39 assay has good accuracy. Additionally, we tested the *EGFR* mutations with abundance from 0.01 to 10%, and the slope between measured and expected abundance was close to 1, indicating that the correlation was strikingly significant (Fig. 3 and Supplementary Figs. S4–7). For L858R/L861Q, S768I, 19Del, T790M and C797S, the LoB was 0.2 copies, while the LoB of 20ins and G719X was 0.3 copies (Supplementary Table S2–S4). Next, we assessed the LoD using serial dilutions of mutant DNA following the CLSI EP17 method. The LoD of L858R/L861Q, 19Del and T790M was 0.339, 0.305 and 0.333 copies/μL, respectively. For rare mutations, the LoD of S768I, G719X, 20ins and C797S was 0.311, 0.434, 0.457 and 0.349 copies/μL, respectively (Supplementary Table S5).

**Table 1:** Analysis accuracy of EGFR L858R, 19Del and T790M in dEGFR39 assay was determined by testing the DNA Reference Standards.

	19Del	L858R	T790M
Expected mutant ratio	12.50%	12.50%	12.50%
Measured mean ± SD	12.63 ± 1.05%	12.13 ± 0.97%	12.19 ± 1.03%
CV	0.08	0.08	0.08
Paired *t*-Test			
* P*-value (vs. Expected)	0.70	0.24	0.36
Significantly different (*P* < 0.05)	NO	NO	NO
Bland–Altman analysis			
Bias ± SD	0.13 ± 1.05%	0.37 ± 0.97%	0.31 ± 1.03%
95% CI	−0.57 to 0.83%	−1.53 to 2.27%	−1.71 to 2.33%

*SD* standard deviation, *CV* coeffiency variation, *CI* confidence interval.

### The consistence between the dEGFR39 method and ARMS-PCR in FFPE tissues

To compare the performance between dEGFR39 assay and ARMS-PCR, 30 FFPE tissues from NSCLC patients were collected. As revealed by the dEGFR39 assay, the individual mutation load ranged from 0.04 to 43.7%. Among these mutations, a large number had an abundance of less than 1% (ranging from 0.04 to 0.79%) (Supplementary Fig. S8).

In this study, we set the respective LoD of mutations as the cut-off value for dEGFR39 assay. Mutations with a concentration less than the LoD value were defined as negative. Consequently, the overall predictive agreement (OPA), positive predictive value (PPV) and negative predictive agreement (NPA) were analysed. In addition, we summarised the detection results of all sites. Mutations with a lower abundance could not be correctly detected using the ARMS-PCR method. In contrast, dEGFR39 was able to detect that the PPV, NPV and OPA were 94.44% (95% CI 74.24–99.01%), 25% (95% CI 8.89–53.23%) and 66.67% (95% CI 48.78–80.77%), respectively (Table 2 and Supplementary Table S6–8). Altogether, these results demonstrate that dEGFR39 can effectively detect low-abundance *EGFR* mutations in patients, and is more sensitive than the ARMS-PCR method.

**Table 2:** Concordance of dEGFR39 for EGFR mutations in FFPE tissues as compared to ARMS.

Mutant type	Patient count				PPA (95% CI)	NPA (95% CI)	OPA (95% CI)
	TP	FN	TN	FP			
L858R/L861Q	15	0	14	1	100 79.61–100%	93.33 70.18–98.81%	96.67 83.33–99.41%
S768I	8	0	19	3	100 67.56–100%	86.36 66.66–95.25%	90 74.38–96.54%
G719X	1	0	29	0	100 20.65–100%	100 88.30–100%	100 88.65–100%
19Del	2	0	28	0	100 34.24–100%	100 87.94–100%	100 88.65–100%
20ins	5	1	19	5	83.33 43.65–96.99%	79.17 59.53–90.76%	80.00 62.69–90.50%
T790M	1	0	26	3	100 20.65–100%	89.66 73.61–96.42%	90 74.38–96.54%
Overall	17	1	3	9	94.44 74.24–99.01%	25 8.89–53.23%	66.67 48.78–80.77%

*TP* true positive, *FN* false negative, *TN* true negative, *FP* false positive, *PPA* positive predict agreement, *NPA* negative predict agreement, *OPA* overall predict agreement, *CI* confidence interval.

### Evaluation of dEGFR39 for detecting *EGFR* mutations in plasma

To evaluate the performance of dEGFR39 in patient-derived plasma, we analysed 33 matched plasma samples from patients with advanced NSCLC. The diagnosis was previously based on the ARMS-PCR method from FFPE tissues, in which 16 patients were positive for *EGFR* mutations (six for L858R/L861Q, eight for 19Del and three for T790M, including compound mutation) and 17 were negative for *EGFR* mutations. The histopathological characteristics of the patients are summarised in Supplementary Table S9. In this analysis, dEGFR39 detected 17 positives and 16 negatives from patient-matched plasma. The PPV, NPV and OPA of *EGFR* mutations were analysed and showed a lower NPA and OPA due to the higher sensitivity of the dEGFR39 assay. Take T790M as an example: two more positive cases were detected by dEGFR39 than ARMS-PCR, reducing its OPA to 93.94% (95% CI 80.39–98.32%), while the OPA of other mutations was 96.97% (Table 3 and Supplementary Table S10–12). This experiment indicates that the dEGFR39 assay is sensitive and specific enough to detect more *EGFR* mutations than other methods.

**Table 3:** Concordance of dEGFR39 for plasma EGFR mutations compared to ARMS.

Mutant type	Patient count				PPA (95% CI)	NPA (95% CI)	OPA (95% CI)
	TP	FN	TN	FP			
L858R/L861Q	6	0	26	1	100 60.97–100%	96.29 81.72–99.34%	96.97 84.68–99.46%
S768I	7	1	25	0	85.71 52.91–97.76%	100 86.68–100%	96.97 84.68–99.46%
G719X	1	0	32	0	100 20.65–100%	100 89.28–100%	100 89.57–100%
19Del	0	0	33	0	NA	100 89.57–100%	100 89.57–100%
20ins	1	0	32	0	100 20.65–100%	100 89.28–100%	100 89.57–100%
T790M	3	0	28	2	100 43.85–100%	93.33 78.68–98.15%	93.94 80.39–98.32%
Overall	15	1	14	3	9375.00 71.67–98.89%	82.35 58.97–93.81%	87.88 72.67–95.18%

*TP* true positive, *FN* false negative, *TN* true negative, *FP* false positive, *PPA* positive predict agreement, *NPA* negative predict agreement, *OPA* overall predict agreement, *CI* confidence interval.

### Correlation between *EGFR* mutations detected by dEGFR39 and response to treatment

**Fig. 4:**
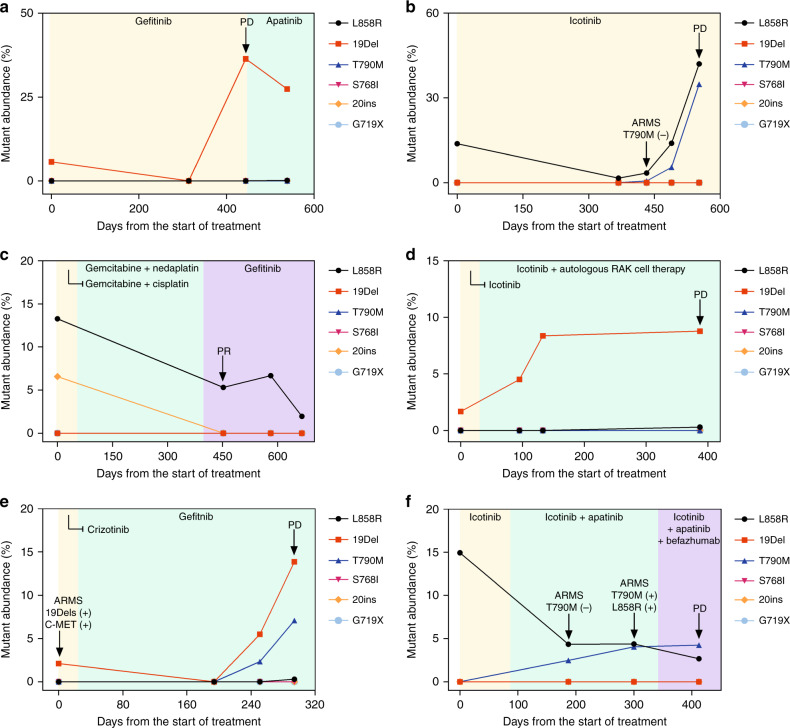
Dynamic detection of EGFR mutations in plasma using a dEGFR39 panel. EGFR frequency of activated mutations decreases initial reception of TKI treatment and subsequently increases (**a**, **e**); T790M mutation was not observed (**c**); emergence of resistant mutation was detected 2 months and 8 months prior to clinical PD, respectively (**b**, **f**), and the activated mutation was never cleared at the beginning of TKI treatment (**d**). The arrow refers to the result of significant changes in the patientʼs tissue samples by ARMS or imaging tests.

We analysed the abundance of *EGFR* mutations before and after TKI treatment. Prior to treatment, multiple plasma samples were collected for the initial assessment. dEGFR39 assay was subsequently performed to monitor the *EGFR* mutation undergoing multiple lines of different treatment (Fig. 4 and Supplementary Table S13–15). Although 20ins and L858R were detected by both dEGFR39 assay and superARMS PCR in patient P-07 at the baseline, there was still a great benefit in taking gefitinib, consistent with the dEGFR39 result at 450 days (Fig. 4c). Patient P-04 received first-line treatment with Icotinib, and was diagnosed as PD at 45 days after treatment, and the subsequent combination therapy was not effective. The mutant abundance of patient P-12, who initially harboured a 19Del mutation, slowly decreased with the treatment of Icotinib, but increased significantly at 444 days (15 months) with the radiographic evidence of PD (Fig. 4a). A steady increase in mutation abundance might indicate poor efficacy of TKI treatment (Fig. 4d). Nevertheless, this data implicate that mutation load as examined by dEGFR39 correlates with disease progression.

### dEGFR39 can predict clinical prognosis after TKI treatment earlier than superARMS PCR

**Fig. 5:**
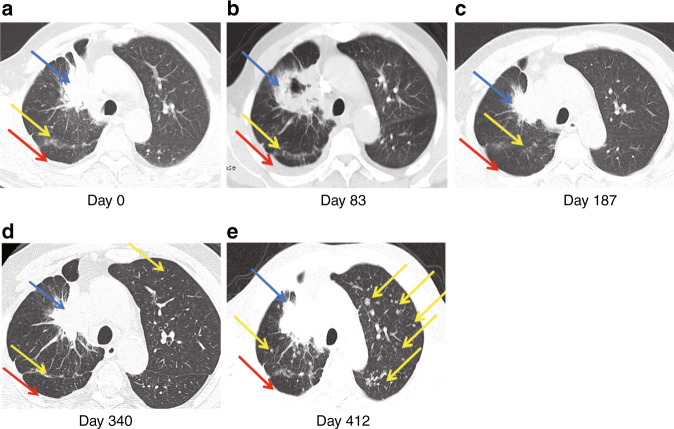
The CT images of patient P-23 are shown in a–e. CT imaging scans performed at the start of Icotinib treatment (day 0, 83), at the change of treatment to Icotinib and Apatinib treatment (day 187, 340), and at the change of treatment to TKI and chemotherapy (day 412). Lesions identified in the lung (blue arrow), liver lobe (yellow arrow), and pleura (red arrow) are indicated.

In NSCLC patients with resistance to TKI treatment, an important mechanism of primary resistance involves a mutation in T790M that blocks the binding of TKI with the adenosine triphosphate domain of *EGFR*.^24,25^ Of the 33 enrolled patients with NSCLC, 13 samples from eight patients were identified by dEGFR39 to harbour the T790M mutation over time; of these, only nine (69%) were detected using SuperARMS. In the plasma of patient P-15, L858R and T790M mutations were simultaneously detected after 368 days of Icotinib treatment. SuperARMS PCR, however, failed to detect the T790M mutation; moreover, there was no significant progress evident in imaging until 121 days (Fig. 4b). Similarly, in patient P-25, dEGFR39 results showed an increase in mutant abundance in 19Del and T790M after 251 days of TKI treatment, which was 43 days ahead of that of imaging progression (Fig. 4e). In patient P-23, the emergence of L858R was initially observed and dropped upon Icotinib treatment. Meanwhile, T790M was detected by dEGFR39, which gradually increased with Icotinib as well as Apatinib plus chemotherapy treatment. In this case, detection of T790M was comparatively delayed by superARMS PCR and CT imaging (Fig. 5). Taken together, this data supports the conclusion that detection of T790M mutations by dEGFR39 occurs relatively earlier than by superARMS PCR and CT imaging, which can play a role in diagnosis and prognosis (Fig. 4f).

## Discussion

Early detection of *EGFR* mutations promises more precise therapy for patients with NSCLC. Using digital PCR, we developed a plasma-based noninvasive method, named dEGFR39, that detects multiple mutations of *EGFR*. To our knowledge, this is the first report regarding the utilisation of digital PCR for this purpose.

Since digital PCR usually has two channels of fluorescence, the system is primarily used for allele mutations in duplex assays because each specialised fluorescent probe can only recognise single allele mutations.^26^ Based on labelling the same fluorescent probe for each mutation, seven common KRAS mutations were consistently detected in plasma from patients with colorectal cancer (CRC).^27^ Moreover, multiple mutations can be recognised by different clusters, which depend on different corresponding concentrations of input primer and probe.^28,29^ However, it remains challenging to identify the cross region of clusters or define the mutation type when the DNA abundance is low.^30^ In contrast, the dEGFR39 assay is ideal for detecting samples with low DNA content, such as plasma. When the template content is too high in the reaction, some additional double-positive signals will appear, which can lead to mistakes in calculating mutation abundance. As shown in Supplementary Fig. S5, due to the large amount of template added, a part of S768I_MU_ and 19Del_MU_ are in one droplet, and the fluorescence intensity of these double-positive signals is different from that of true 19Del_WT_. Therefore, it is clearly divided into two regions, wherein the signal enclosed by the red dotted line is considered to be generated by S768I_MU_ and 19Del_MU_ template amplification. Furthermore, according to the formula provided in this method, the lower the concentration within the confidence range, the lower the frequency of false double-positive signals. The dEGFR39 assay takes advantage of the low abundance of DNA in plasma, making the test results more accurate.

Another study reported a drop-off method to achieve multiple detections; although this method can detect mutations located together, it fails to simultaneously detect other mutation types.^26^ To overcome these challenges, we have devised a three step reaction by optimising annealing temperature, combined probe concentrations, and modifying system configuration with fluorescent compensation (Supplementary Fig. S9). After these improvements, dEGFR39 exhibits superior performance to profile the driver mutations of the *EGFR* gene. It is important to note that our data show that accuracy for dEGFR39 from plasma and ARMS from FFPE is 87.88%, which is similar to previous studies (80.8–94.19%).^31–33^

Importantly, it has been previously reported that digital PCR has technical issues such as the phenomenon of “rain”, which ranges between explicit positive and negative droplets. This issue eventually hinders the correct setting of threshold and consequently leads to failure of the digital PCR experiment.^34,35^ In this study, we induced a “double positive” discrimination method in the detection of 19Del, 20ins and C797S, which made the distribution of rain primarily in the diagonal area in the 2D plot diagram, thus avoiding interference with the identification of positive droplets. This approach greatly facilitated multiplex detection and led to less background noise (Fig. 2). Although it was minimal, the rain phenomenon still occurred. While detecting 20ins mutant locus from patient-derived FFPE tissues, we observed an increase in LoB and low correlation with the accuracy of 66.67% (95% CI 48.78–80.77%). Interestingly, while using patient-derived plasma, we observed a significant improvement in the ratio of signal-to-noise. We reason that FFPE, but not plasma, might potentially contain an inhibitor for digital PCR analysis.

Several studies have demonstrated that mutant abundance of *EGFR* is closely associated with the response to treatment.^36^ More importantly, dEGFR39 is more sensitive than superARMS PCR and CT imaging, thus allowing early detection of *EGFR* mutations. Of note, some patients, although positive for *EGFR* driver mutations, received no clinical benefit from EGFR-TKI treatment (Fig. 4). In these patients, we observed an obvious trend of increasing mutation load.

Another comparative advantage of the dEGFR39 assay is low sample input. Whereas the commercial *EGFR* kit (ARMS method) requires detection of all *EGFR* mutations in eight tubes^37^, dEGFR39 uses three reactions to sufficiently characterise the *EGFR* mutation status even from plasma-derived DNA. In future studies, assessment of *EGFR*, *ALK*, *ROS1* and perhaps other oncogenes can be streamlined in the same digital PCR platform. Although NGS can parallelly analyse multiple variations, including unknown variations, digital PCR is still the most suitable method in clinical testing, because of its higher sensitivity, easier-to-understand results, low turn-around time and low cost.

In conclusion, we developed a noninvasive plasma-based digital PCR method, hereby named dEGFR39, that allows simultaneous detection of multiple mutation sites of the *EGFR* gene. In this report, we provide evidence that this method is highly sensitive, reliable and cost-efficient, promising efficacy for clinical diagnosis and treatment assessment for patients with NSCLC.

## Supplementary information

Supplementary Information

## Data Availability

The datasets used and/or analysed during the current study are available from the corresponding author on reasonable request.
