# Supplementary material for: Plasma-based early screening and monitoring of EGFR mutations in NSCLC patients by a 3-color digital PCR assay
Source: Br J Cancer. 2020 Aug 12;123(9):1437–44. doi: 10.1038/s41416-020-1024-2 (PMC7592053; doi:10.1038/s41416-020-1024-2)
Supplement: Supplementary file 1 — Supplementary Information [file 41416_2020_1024_MOESM1_ESM.pdf]

Supplementary

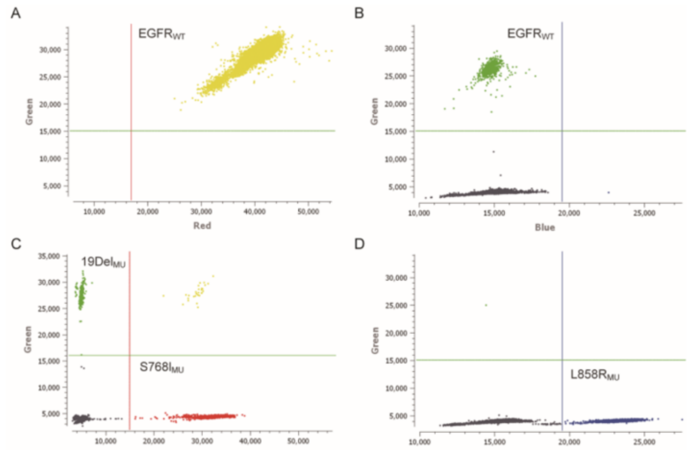

Fig. S1. The specificity of EGFR L858R, S768I and 19Del within the dEGFR39 assay. The specific detection is completed in two PCR wells, with both reactions containing all primer probes. (A) and (B) are 2D images of dPCR reactions containing only wild-type DNA; (C) and (D) are 2D images of dPCR reactions containing only L858R, S768I and 19Del mutant plasmids.

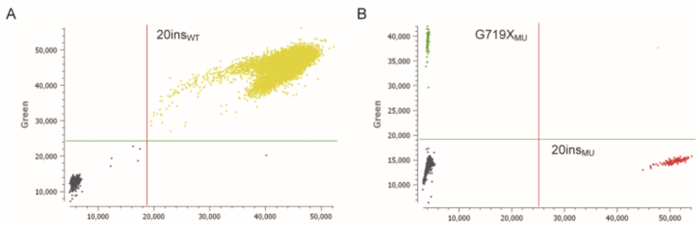

Fig. S2. The specificity of EGFR G719X and 20ins within dEGFR39 assay in a 2D plot. The specific detection is completed in two PCR wells, with both reactions containing all primer probes. (A) is a 2D image of dPCR reactions containing only wild-type DNA; (B) is a 2D image of dPCR reactions containing only G719X and 20ins mutant plasmids.

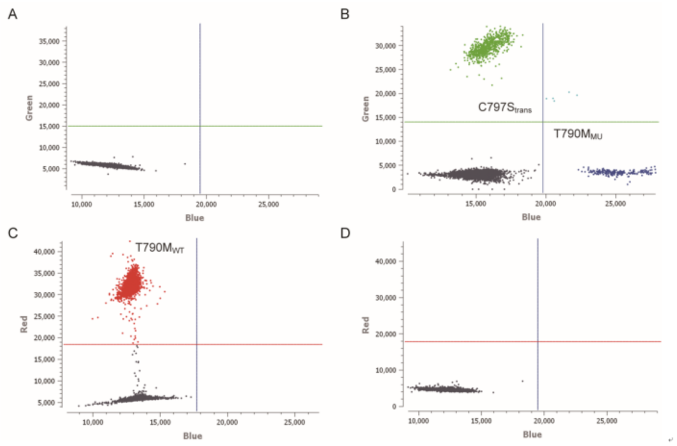

Fig. S3. The specificity of EGFR T790M and C797S within dEGFR39 assay in a 2D plot. The specific detection is completed in two PCR wells, with both reactions containing all primer probes. (A) and (B) are 2D images of dPCR reactions containing only wild-type DNA; (C) and (D) are 2D images of dPCR reactions containing only T790M and C797S mutant plasmids.

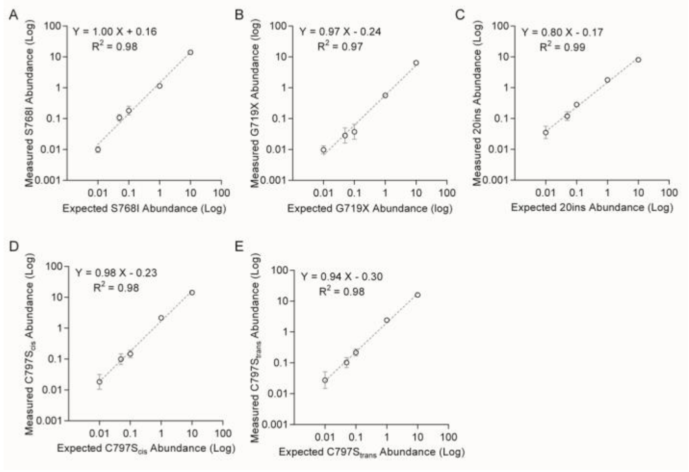

Fig. S4. The linearity of EGFR S768I, 20ins, G719X and C797S in dEGFR39 assay. EGFR mutations were detected on a series of DNA with a mutant ratio of 10%, 1%, 0.1%, 0.05% and 0.01%. A regression plot for the dilutions shows linearity and a good correlation for expected and measured values.

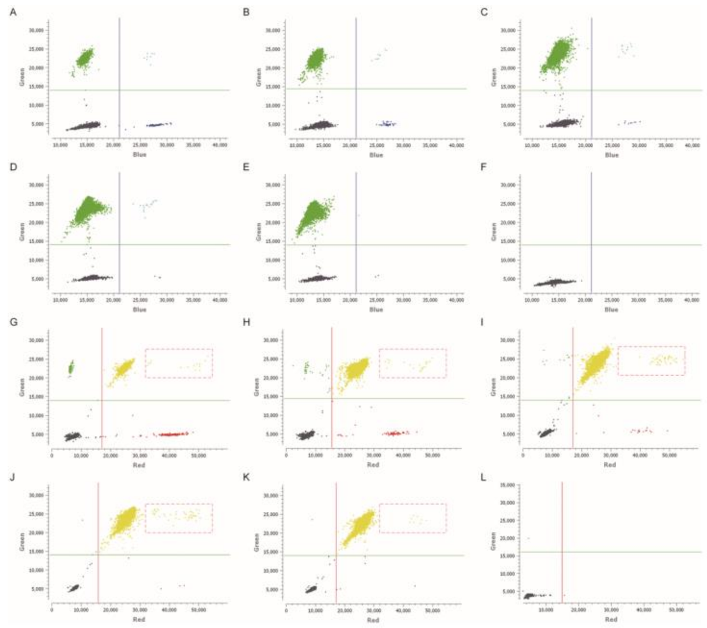

Fig. S5. The sensitivity of EGFR L858R, S768I and 19Del within dEGFR39 assay in a 2D plot. EGFR mutations were detected on a series of DNA with a mutant ratio of 10%, 1%, 0.1%, 0.05%, 0.01% and NTC. (A) to (F) are the dPCR results in 2D dot-plots of HEX (Green) and FAM (Blue) channels; (G) to (L) are the dPCR results in 2D dot-plots of HEX(Green) and CY5(Red) channels.

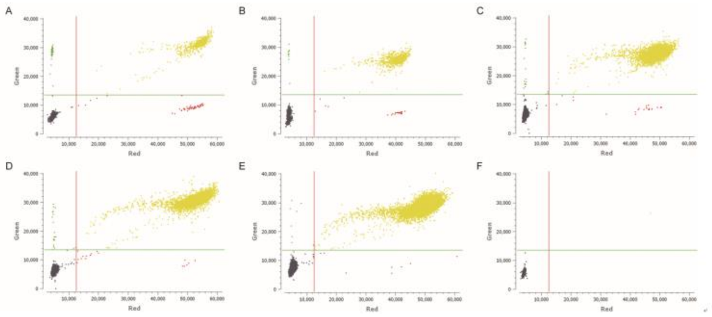

Fig. S6. The sensitivity of EGFR G719X and 20ins within dEGFR39 assay in a 2D plot. (A) to (F) are the dPCR results of HEX(Green) and CY5(Red) channels with a mutant ratio of 10%, 1%, 0.1%, 0.05%, 0.01% and NTC.

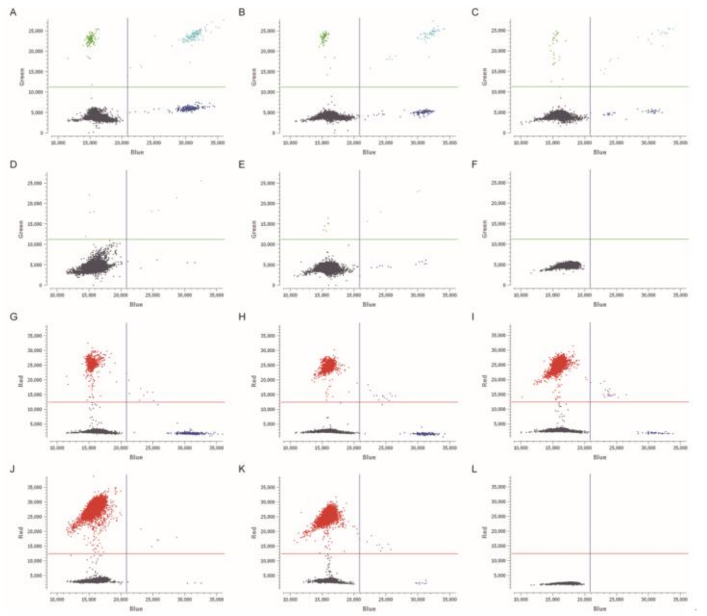

Fig. S7. The sensitivity of EGFR T790M and C797S within dEGFR39 assay in a 2D plot. EGFR mutations were detected on a series of DNA with a mutant ratio of 10%, 1%, 0.1%, 0.05%, 0.01% and NTC. (A) to (F) are the dPCR results in 2D dot-plots of HEX (Green) and FAM (Blue) channels; (G) to (L) are the dPCR results in 2D dot-plots of FAM(Blue) and CY5(Red) channels.

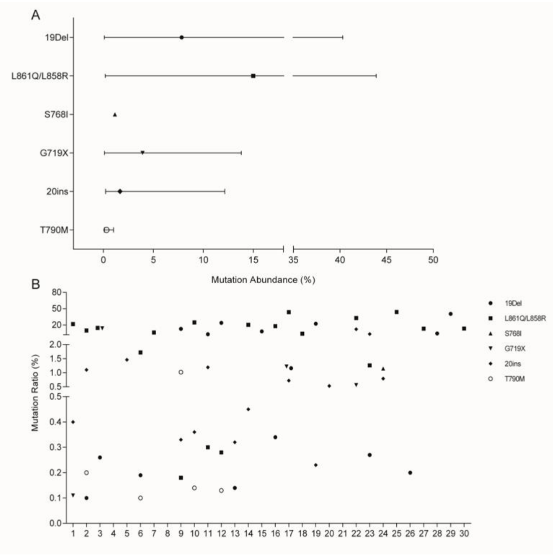

Fig. S8. Longitudinal analysis of mutant abundance in 30 NSCLC patients.

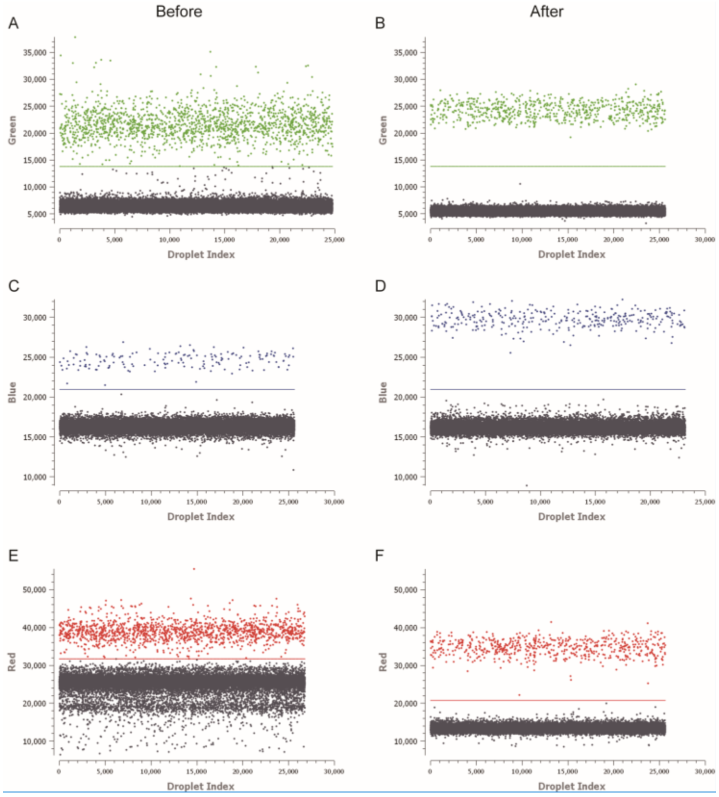

Fig. S9. Optimisation of signal partition in a multiplex dPCR assay. Optimisation of dEGFR39 assay for annealing temperature are shown in (A) and (B), concentrations in (C) and (D), and combinations in (E) and (F).

Table S1. The locus information of dEGFR39 assay.

Exon	Amino acid change	Mutation site	COSMIC	Sequence
Exon18	c.2155G>A	p.G719S	6252	CTTTCCAGCATGGTGAGGGCTGAGGTGACCCTTGTCTCTGTGTTCTTGTCCCCCCCAGCTTGTGGAGCCTCTTACACCCAGTGGAGAAGCTCCCAACCAAGCTCTCTTGAGGATCTTGAAGGAAACTGAATTCAAAAAGATCAAAGTGCT GAGCTCCGGTGCGTTCGGCACGGTGTATAAGGTAAGGTCCCTGGCACAGGCCTCTGGGCTGGGCCGCAGGGCCTCTCATGGTCTGGTGGGGAGCCCAGAGTCCTTGCAAGCTGTATATTTCCATCATCTACTTTACTCTTTGTTTCACTGA
Exon18	c.2155G>T	p.G719C	6253	CTTTCCAGCATGGTGAGGGCTGAGGTGACCCTTGTCTCTGTGTTCTTGTCCCCCCCAGCTTGTGGAGCCTCTTACACCCAGTGGAGAAGCTCCCAACCAAGCTCTCTTGAGGATCTTGAAGGAAACTGAATTCAAAAAGATCAAAGTGCT GTGCTCCGGTGCGTTCGGCACGGTGTATAAGGTAAGGTCCCTGGCACAGGCCTCTGGGCTGGGCCGCAGGGCCTCTCATGGTCTGGTGGGGAGCCCAGAGTCCTTGCAAGCTGTATATTTCCATCATCTACTTTACTCTTTGTTTCACTGA
Exon18	c.2156G>C	p.G719A	6239	CTTTCCAGCATGGTGAGGGCTGAGGTGACCCTTGTCTCTGTGTTCTTGTCCCCCCCAGCTTGTGGAGCCTCTTACACCCAGTGGAGAAGCTCCCAACCAAGCTCTCTTGAGGATCTTGAAGGAAACTGAATTCAAAAAGATCAAAGTGCT GGCCTCCGGTGCGTTCGGCACGGTGTATAAGGTAAGGTCCCTGGCACAGGCCTCTGGGCTGGGCCGCAGGGCCTCTCATGGTCTGGTGGGGAGCCCAGAGTCCTTGCAAGCTGTATATTTCCATCATCTACTTTACTCTTTGTTTCACTGA
Exon18	c.2156G>A	p.G719D	18425	CTTTCCAGCATGGTGAGGGCTGAGGTGACCCTTGTCTCTGTGTTCTTGTCCCCCCCAGCTTGTGGAGCCTCTTACACCCAGTGGAGAAGCTCCCAACCAAGCTCTCTTGAGGATCTTGAAGGAAACTGAATTCAAAAAGATCAAAGTGCT GGACTCCGGTGCGTTCGGCACGGTGTATAAGGTAAGGTCCCTGGCACAGGCCTCTGGGCTGGGCCGCAGGGCCTCTCATGGTCTGGTGGGGAGCCCAGAGTCCTTGCAAGCTGTATATTTCCATCATCTACTTTACTCTTTGTTTCACTGA
Exon19	c.2240_2251 del 12	p.L747_T751>S	6210	CATGGACAGCCCCAGTGTCCCTCACCTTCGGGGTGCATCGCTGGTAACATCCACCCAGATCACTGGGCAGCATGTGGCACCATCTCACAATTGCCAGTTAACGTCTTCCTTCTCTCTCTGTCATAGGGACTCTGGATCCCAGAAGGTGAGAA AGTTAAAATTCCCGTCGCTATCAAGGAATCATCTCCGAAAGCCAACAAGGAAATCCTCGATGTGAGTTTCTGCTTTGCTGTGTGGGGGTCCATGGCTCTGAACCTCAGGCCCACCTTTTCTCATGTCTGGC
Exon19	c.2240_2254 del 15	p.L747_T751delLREAT	12369	
Exon19	c.2240_2257 del 18	p.L747_P753>S	12370	
Exon19	c.2237_2257>TCT	p.E746_P753>VS	18427	
Exon19	c.2238_2252del15	p.L747_T751delLREAT	23571	
Exon19	c.2239_2247 del 9	p.L747_E749del	6218	CATGGACAGCCCCAGTGTCCCTCACCTTCGGGGTGCATCGCTGGTAACATCCACCCAGATCACTGGGCAGCATGTGGCACCATCTCACAATTGCCAGTTAACGTCTTCCTTCTCTCTCTGTCATAGGGACTCTGGATCCCAGAAGGTGAGAA AGTTAAAATTCCCGTCGCTATCAAGGAAGCAACATCTCCGAAAGCCAACAAGGAAATCCTCGATGTGAGTTTCTGCTTTGCTGTGTGGGGGTCCATGGCTCTGAACCTCAGGCCCACCTTTTCTCATGTCTGGC
Exon19	c.2238_2255 del 18	p.E746_S752>D	6220	
Exon19	c.2235_2249 del 15	p.E746_A750delELREA	6223	
Exon19	c.2236_2250 del 15	p.E746_A750delELREA	6225	
Exon19	c.2239_2253 del 15	p.L747_T751del	6254	
Exon19	c.2239_2256 del 18	p.L747_S752delLREATS	6255	
Exon19	c.2237_2254 del 18	p.E746_S752>A	12367	
Exon19	c.2237_2255>T	p.E746_S752>V	12384	
Exon19	c.2239_2256>CAA	p.L747_S752>Q	12403	
Exon19	c.2237_2253>TTGCT	p.E746_T751>VA	12416	
Exon19	c.2235_2255>AAT	p.E746_S752>I	12385	
Exon19	c.2237_2251 del 15	p.E746_T751>A	12678	
Exon19	c.2236_2253 del 18	p.E746_T751delELREAT	12728	
Exon19	c.2233_2247del15	p.K745_E749delKELRE	26038	
Exon19	c.2237_2252>T	p.E746_T751>V	12386	
Exon19	c.2239_2258>CA	p.L747_P753>Q	12387	CATGGACAGCCCCAGTGTCCCTCACCTTCGGGGTGCATCGCTGGTAACATCCACCCAGATCACTGGGCAGCATGTGGCACCATCTCACAATTGCCAGTTAACGTCTTCCTTCTCTCTCTGTCATAGGGACTCTGGATCCCAGAAGGTGAGAA AGTTAAAATTCCCGTCGCTATCAAGGAGCCAACATCTCCGAAAGCCAACAAGGAAATCCTCGATGTGAGTTTCTGCTTTGCTGTGTGGGGGTCCATGGCTCTGAACCTCAGGCCCACCTTTTCTCATGTCTGGC
Exon19	c.2239_2248 TTAAGAGAAG>C	p.L747_ A750>P	12382	
Exon19	c.2239_2251>C	p.L747_T751>P	12383	
Exon19	c.2238_2252>GCA	p.L747_T751>Q	12419	
Exon19	c.2238_2248>GC	p.L747_A750>P	12422	
Exon19	c.2235_2248>AATTC	p.E746_A750>IP	13550	
Exon19	c.2235_2252>AAT	p.E746_T751>I	13551	
Exon19	c.2235_2251>AATTC	p.E746_T751>IP	13552	
Exon20	c.2369C>T	p.T790M	6240	ACACTGACGTGCCTCTCCCTCCCTCCAGGAAGCCTACGTGATGGCCAGCGTGGACAACCCCCACGTGTGCCGCCTGCTGGGCATCTGCCTCACCTCCACCGTGCAGCTCATCATGCAGCTCATGCCCTTCGGCTGCCTCCTGGACTATGTC CGGGAACACAAAGACAATATTGGCTCCCAGTACCTGCTCAACTGGTGTGTGCAGATCGCAAAGGTAATCAGGGAAGGGAGATACGGGGAGG
Exon20	c.2303G>T	p.S768I	6241	CCATGGGTATTTGAAACTCAAGATCGCATTCATGCGTCTTCACCTGGAAGGGGTCCATGTGCCCCTCCTTCTGGCCACCATGCGAAGCCACACTGACGTGCCTCTCCCTCCCTCCAGGAAGCCTACGTGATGGCCATCGTGGACAACCCC CACGTGTGCCGCCTGCTGGGCATCTGCCTCACCTCCACCGTGCAGCTCATCACGCAGCTCATGCCCTTCGGCTGCCTCCTGGACTATGTCCGGGAACACAAAGACAATATTGGCTCCCAGTACCTGCTCAACCATGG
Exon20	c.2309_2310AC>CCAGCGTGGAT	p.V769_D770insASV	13558	CCATGGTTCACCTGGAAGGGGTCCATGTGCCCCTCCTTCTGGCCACCATGCGAAGCCACACTGACGTGCCTCTCCCTCCCTCCAGGAAGCCTACGTGATGGCCAGCGTGGCCAGCGTGGATAACCCCCACGTGTGCCGCCTGCTGGGCA TCTGCCTCACCTCCACCGTGCAGCTCATCACGCAGCTCATGCCCTTCGGCTGCCTCCTGGACTATGTCCGGGAACACAAAGACAATATTGGCTCCCAGTACCTGCTCAACTGGTGTGTGCAGATCGCAAAGGTAATCAGGGAAGGGAGATA CGGGGAGGGGAGATAAGGAGCCAGGATCCTCACATGCGGTCTGCGCTCCTGGGATAGCAACCATGG
Exon20	c.2310_2311insGGT	p.D770_N771insG	12378	CCATGGTTCACCTGGAAGGGGTCCATGTGCCCCTCCTTCTGGCCACCATGCGAAGCCACACTGACGTGCCTCTCCCTCCCTCCAGGAAGCCTACGTGATGGCCAGCGTGGACGGTAACCCCCACGTGTGCCGCCTGCTGGGCATCTGCCT CACCTCCACCGTGCAGCTCATCACGCAGCTCATGCCCTTCGGCTGCCTCCTGGACTATGTCCGGGAACACAAAGACAATATTGGCTCCCAGTACCTGCTCAACTGGTGTGTGCAGATCGCAAAGGTAATCAGGGAAGGGAGATACGGGGA GGGGAGATAAGGAGCCAGGATCCTCACATGCGGTCTGCGCTCCTGGGATAGCAACCATGG
Exon20	C.2319_2320INSCAC	P.H773_V774INSH	12377	CCATGGTTCACCTGGAAGGGGTCCATGTGCCCCTCCTTCTGGCCACCATGCGAAGCCACACTGACGTGCCTCTCCCTCCCTCCAGGAAGCCTACGTGATGGCCAGCGTGGACAACCCCCACCACGTGTGCCGCCTGCTGGGCATCTGCCT CACCTCCACCGTGCAGCTCATCACGCAGCTCATGCCCTTCGGCTGCCTCCTGGACTATGTCCGGGAACACAAAGACAATATTGGCTCCCAGTACCTGCTCAACTGGTGTGTGCAGATCGCAAAGGTAATCAGGGAAGGGAGATACGGGGA GGGGAGATAAGGAGCCAGGATCCTCACATGCGGTCTGCGCTCCTGGGATAGCAACCATGG
Exon21	c.2573T>G	p.L858R	6224	TTGGAGGACCGTCGCTTGGTGCACCGCGACCTGGCAGCCAGGAACGTACTGGTGAAAACACCGCAGCATGTCAAGATCACAGATTTTGGGCGGGCCAAACTGCTGGGTGCGGAAGAGAAAGAATACCATGCAGAAGGAGGCAAAGT AAGGAGGTGGCTTTAGGTCAGCCAGCATTTTCCTGACACCAGGGACCAGGCTGC
Exon21	c.2582T>A	p.L861Q	6213	CCATGGCAGGGCATGAACTACTTGGAGGACCGTCGCTTGGTGCACCGCGACCTGGCAGCCAGGAACGTACTGGTGAAAACACCGCAGCATGTCAAGATCACAGATTTTGGGCTGGCCAAACAGCTGGGTGCGGAAGAGAAAGAATAC CATGCAGAAGGAGGCAAAGTAAGGAGGTGGCTTTAGGTCAGCCAGCATTTTCCTGACACCAGGGACCAGGCTGCCTTCCCACTAGCTGTCCATGG

Table S2. Analytical limit of blank (LoB) of dEGFR39 assay-#1 (L858R/L861Q/19del/S768I)

Num	Total Number of droplets	L858R/L861Q			19Del					S768I		
		Concentration of L858R$_{MU}$/ L861Q$_{MU}$ (copies/μl)	Number of positive droplets	Abundance	Concentration of 19Del$_{MU}$ (copies/μl)	Number of positive droplets	Concentration of EGFR$_{WT}$ (copies/μl)	Number of positive droplets	Abundance	Concentration of S768I (copies/μl)	Number of positive droplets	Abundance
	24513	0.07	1	**0.0021%**	0.14	2	3385.66	21034	**0.0041%**	0.1	2	0.0041%
	21457	0.00	0	**0.0000%**	0.00	0	3489.57	18582	**0.0000%**	0.0	0	0.0000%
	22368	0.08	1	**0.0023%**	0.08	1	3291.53	19012	**0.0023%**	0.1	1	0.0023%
	25478	0.13	2	**0.0039%**	0.00	0	3404.90	21904	**0.0000%**	0.1	1	0.0020%
	26987	0.00	0	**0.0000%**	0.00	0	3038.65	22316	**0.0000%**	0.2	3	0.0062%
	24513	0.07	1	**0.0023%**	0.07	1	2973.82	20103	**0.0023%**	0.1	1	0.0023%
	23235	0.22	3	**0.0075%**	0.29	4	2942.22	18975	**0.0100%**	0.3	4	0.0100%
	26541	0.26	4	**0.0082%**	0.06	1	3118.95	22154	**0.0021%**	0.1	2	0.0041%
	25741	0.07	1	**0.0021%**	0.13	2	3208.30	21698	**0.0041%**	0.0	0	0.0000%
	26985	0.00	0	**0.0000%**	0.00	0	3194.65	22716	**0.0000%**	0.1	1	0.0020%
	26457	0.06	1	**0.0018%**	0.06	1	3531.29	23008	**0.0018%**	0.1	2	0.0037%
	21547	0.00	0	**0.0000%**	0.08	1	3125.89	17989	**0.0025%**	0.0	0	0.0000%
	23548	0.14	2	**0.0046%**	0.07	1	3170.42	19763	**0.0023%**	0.0	0	0.0000%
	26134	0.00	0	**0.0000%**	0.20	3	3685.17	23015	**0.0053%**	0.1	1	0.0018%
	23654	0.14	2	**0.0046%**	0.07	1	3106.19	19709	**0.0023%**	0.1	1	0.0023%
	25854	0.00	0	**0.0000%**	0.07	1	3226.57	21836	**0.0020%**	0.1	2	0.0041%
	24198	0.07	1	**0.0020%**	0.07	1	3504.00	20989	**0.0020%**	0.2	3	0.0060%
	25471	0.20	3	**0.0063%**	0.00	0	3184.68	21415	**0.0000%**	0.1	1	0.0021%
	23654	0.07	1	**0.0022%**	0.14	2	3283.14	20091	**0.0044%**	0.0	0	0.0000%
	25478	0.07	1	**0.0022%**	0.07	1	3060.10	21119	**0.0022%**	0.0	0	0.0000%
MEAN		0.08			0.08					0.09		
SD		0.078219			0.073543					0.081093		
LoB		0.212			0.201					0.219		

Table S3. Analytical limit of blank (LoB) of dEGFR39 assay-#2 (G719X&20ins)

Num	Total Number of droplets	G719X					20Ins				
		Concentration of G719X$_{MU}$ (copies/μl)	Number of positive droplets	Concentration of G719X$_{WT}$ (copies/μl)	Number of positive droplets	G719X%	Concentration of 20ins$_{MU}$ (copies/μl)	Number of positive droplets	Concentration of EGFR$_{WT}$ (copies/μl)	Number of positive droplets	20ins%
	25134	0.14	2	2262.09	18456	0.0060%	0.36	5	2759.74	20145	0.0131%
	22354	0.23	3	2472.00	17102	0.0093%	0.31	3	2792.82	18002	0.0109%
	25436	0.07	1	2104.26	18023	0.0032%	0.20	3	2671.45	20119	0.0075%
	24451	0.28	4	2098.51	17301	0.0133%	0.14	2	2749.37	19568	0.0051%
	26415	0.00	0	2175.64	19032	0.0000%	0.19	3	2744.27	21124	0.0071%
	24513	0.14	2	2293.56	18119	0.0061%	0.14	2	2724.58	19546	0.0051%
	26454	0.06	1	2156.11	18975	0.0030%	0.00	0	3100.75	22154	0.0000%
	26457	0.19	3	2211.26	19215	0.0088%	0.26	3	2750.86	21178	0.0094%
	27846	0.06	1	2205.50	20198	0.0028%	0.00	0	2722.88	22198	0.0000%
	23145	0.00	0	2518.73	17854	0.0000%	0.20	2	3174.52	19542	0.0062%
	21365	0.16	2	2427.91	16214	0.0066%	0.16	2	2946.63	17564	0.0054%
	25648	0.13	2	2162.74	18425	0.0062%	0.09	1	2313.85	19037	0.0038%
	26548	0.13	2	2328.37	19763	0.0055%	0.00	0	2391.40	20009	0.0000%
	23644	0.07	1	2445.51	18002	0.0030%	0.00	0	2989.55	19542	0.0000%
	23655	0.14	2	2340.45	17652	0.0062%	0.14	2	2754.81	18946	0.0052%
	26415	0.26	4	2422.44	20026	0.0107%	0.13	2	2652.90	20833	0.0049%

	21546	0.08	1	2396.22	16254	0.0033%	0.21	2	2753.68	17254	0.0077%
	25416	0.20	3	2337.25	18954	0.0086%	0.00	0	2593.22	19854	0.0000%
	23564	0.43	6	2342.42	17591	0.0186%	0.07	1	2655.14	18591	0.0027%
	24751	0.07	1	2220.62	18013	0.0031%	0.09	1	2785.99	19913	0.0033%
MEAN		0.14					0.13				
SD		0.10388					0.105781				
LOB		0.313					0.309				

Table S4. Analytical limit of blank (LoB) of dEGFR39 assay-#3 (T790M/C797S)

Num	Total Number of droplets	T790M					$C797S_{TRANS}$			$C797S_{cis}$		
		Concentration of $T790M_{Mu}$ (copies/µl)	Number of positive droplets	Concentration of $T790M_{WT}$ (copies/µl)	Number of positive droplets	$T790M_{Mu}$%	Concentration of $C797S_{TRANS}$ (copies/µl)	Number of positive droplets	$C797S_{TRANS}$%	Concentration of C797Scis (copies/µl)	Number of positive droplets	$C797S_{TRANS}$%
	28139	0.14	2	2948.54	20156	0.0047%	0.07	1	0.0024%	0.00	0	0.0000%
	23548	0.14	2	3161.41	19854	0.0046%	0.07	1	0.0023%	0.08	1	0.0027%
	24254	0.07	1	2921.49	19875	0.0024%	0.00	0	0.0000%	0.10	1	0.0035%
	24368	0.00	0	2771.41	19564	0.0000%	0.07	1	0.0025%	0.16	2	0.0058%
	26541	0.00	0	2772.51	21312	0.0000%	0.06	1	0.0023%	0.16	2	0.0059%
	25412	0.00	0	2688.88	20154	0.0000%	0.00	0	0.0000%	0.09	1	0.0034%
	25538	0.13	2	2619.60	20035	0.0051%	0.07	1	0.0026%	0.08	1	0.0029%
	25417	0.00	0	3027.35	21104	0.0000%	0.00	0	0.0000%	0.27	3	0.0089%
	24785	0.07	1	2762.77	19874	0.0025%	0.07	1	0.0025%	0.15	2	0.0053%
	26541	0.06	1	2718.86	21145	0.0024%	0.06	1	0.0024%	0.08	1	0.0029%
	25465	0.20	3	2651.07	20078	0.0076%	0.20	3	0.0076%	0.00	0	0.0000%
	24351	0.00	0	2940.82	20004	0.0000%	0.07	1	0.0024%	0.00	0	0.0000%
	25741	0.00	0	2892.54	21014	0.0000%	0.07	1	0.0023%	0.00	0	0.0000%
	25644	0.13	2	2897.32	20948	0.0046%	0.13	2	0.0046%	0.00	0	0.0000%
	23655	0.22	3	2938.30	19426	0.0074%	0.22	3	0.0074%	0.00	0	0.0000%
	21245	0.08	1	3230.77	18045	0.0025%	0.08	1	0.0025%	0.09	1	0.0028%
	22543	0.08	1	2733.13	17998	0.0028%	0.08	1	0.0028%	0.14	1	0.0051%
	23254	0.29	4	2880.70	18954	0.0102%	0.07	1	0.0025%	0.21	2	0.0074%
	24152	0.07	1	2844.80	19591	0.0025%	0.14	2	0.0050%	0.17	2	0.0059%
	25241	0.07	1	2687.13	20013	0.0025%	0.07	1	0.0025%	0.17	2	0.0062%
MEAN		0.09					0.08			0.10		
SD		0.082776					0.056485			0.080589		
LoB		0.224					0.173			0.230		

Table S5. LoD calculation using probit regression analysis.

		L858R / L861Q	E19 Del	S768I	G719X	E20 ins	T790M	C797S
1 fold LoB	Concentration (copies/µL)	0.212	0.201	0.219	0.312	0.309	0.224	0.173
	% Positive	75%	60%	65%	70%	55%	70%	65%
	SD	0.09	0.073	0.086	0.078	0.091	0.067	0.072
1.5 fold LoB	% Positive	95%	95%	100%	100%	100%	95%	95%
	SD	0.077	0.063	0.056	0.074	0.09	0.066	0.107
2 fold LoB	% Positive	100%	100%	100%	100%	100%	100%	100%
	SD	0.037	0.026	0.037	0.026	0.036	0.025	0.026
3 fold LoB	% Positive	100%	100%	100%	100%	100%	100%	100%
	SD	0.046	0.022	0.033	0.021	0.035	0.027	0.022
**LoD** (copies/µL)		0.339	0.305	0.311	0.434	0.457	0.333	0.349

Table S6. FFPE samples detection with dEGFR39 assay-#1 (L858R/L861Q/19del/S768I)

Num	ARMS			Total Number of droplets	L858R/L861Q				19Del							S768I			
	L858R / L861Q	E19 del	S768I		Concentration of $L858R_{MU}$/ $L861Q_{MU}$ (copies/µl)	Number of positive droplets	Relative uncertainty	Abundance	Concentration of $19Del_{MU}$ (copies/µl)	Number of positive droplets	Relative uncertainty	Concentration of 19Del $_{Ref}$ (copies/µl)	Number of positive droplets	Relative uncertainty	Abundance	Concentration of S768I (copies/µl)	Number of positive droplets	Relative uncertainty	Abundance
CZ-01	√			26360	32.4	496	9%	**21.61%**	0.06	1	196%	149.9	2216	4%	**0.04%**	0.1	1	196.00%	0.04%
CZ-02	√			26314	24.4	374	10%	**9.81%**	0.26	4	98%	248.5	3566	3%	**0.10%**	0.0	0	inf	0.00%
CZ-03	√			25325	15.2	224	13%	**14.61%**	0.27	4	98%	103.8	1494	5%	**0.26%**	0.0	0	inf	0.00%
CZ-04			√	24427	0.07	1	196%	**0.06%**	0	0	inf	109.1	1513	5%	**0.00%**	6.4	91	20.55%	5.84%
CZ-05				23948	0.14	2	139%	**0.16%**	0	0	inf	87.2	1193	6%	**0.00%**	0.0	0	inf	0.00%
CZ-06	√			25643	4.93	74	23%	**1.72%**	0.53	8	69%	285.6	3951	3%	**0.19%**	0.0	0	inf	0.00%
CZ-07	√			26204	3.06	47	29%	**6.09%**	0.07	1	196%	50.2	760	7%	**0.14%**	0.1	1	196.00%	0.13%
CZ-08		√		20962	0.33	4	98%	**0.18%**	23.7	289	12%	161.4	1891	5%	**12.80%**	0.0	0	inf	0.00%
CZ-09	√			24605	33.8	483	9%	**24.75%**	0.14	2	139%	136.4	1890	5%	**0.10%**	0.0	0	inf	0.00%
CZ-10		√		26630	0.32	5	88%	**0.30%**	3.14	49	28%	102.9	1558	5%	**2.96%**	0.2	3	113.16%	0.19%
CZ-11		√		24498	0.56	8	69%	**0.28%**	47.5	672	8%	152	2087	4%	**23.81%**	0.0	0	inf	0.00%
CZ-12				25888	0.13	2	139%	**0.04%**	0.4	6	80%	292	4071	3%	**0.14%**	0.0	0	inf	0.00%
CZ-13	√			26597	15.3	238	13%	**20.29%**	0	0	inf	75.4	1150	6%	**0.00%**	0.0	0	inf	0.00%
CZ-14		√		25230	0.07	1	196%	**0.06%**	9.97	147	16%	112	1602	5%	**8.17%**	0.1	1	196.00%	0.06%
CZ-15	√			24219	29.4	413	10%	**17.87%**	0.56	8	69%	164	2219	4%	**0.34%**	0.1	1	196.00%	0.04%
CZ-16	√	√		25536	20	297	11%	**43.70%**	0.53	6	80%	45.5	672	8%	**1.16%**	0.1	1	196.00%	0.15%
CZ-17	√			25015	9.51	139	17%	**4.01%**	0.2	3	113%	236.7	3239	3%	**0.08%**	0.0	0	inf	0.00%
CZ-18		√		26468	0.06	1	196%	**0.05%**	29.7	456	9%	103.3	1554	5%	**22.33%**	0.0	0	inf	0.00%
CZ-19				26602	0.13	2	139%	**0.12%**	0.13	2	139%	111.7	1685	5%	**0.12%**	0.0	0	inf	0.00%
CZ-20				26032	0.2	3	113%	**0.07%**	0.2	3	113%	297.7	4167	3%	**0.07%**	0.1	1	196.00%	0.02%
CZ-21	√			23849	18.9	263	12%	**32.73%**	0.14	2	139%	57.6	792	7%	**0.24%**	0.0	0	inf	0.00%
CZ-22	√			24680	0.97	14	52%	**1.26%**	0.21	3	113%	76.7	1084	6%	**0.27%**	0.0	0	inf	0.00%
CZ-23				26103	0.07	1	196%	**0.12%**	0.07	1	196%	60.4	908	7%	**0.12%**	0.1	1	196.00%	0.11%
CZ-24	√			26401	40.4	617	8%	**43.88%**	0.06	1	196%	92	1385	5%	**0.07%**	0.0	0	inf	0.00%
CZ-25				24966	0.07	1	196%	**0.05%**	0.27	4	98%	133.5	1879	5%	**0.20%**	0.0	0	inf	0.00%
CZ-26	√			25124	7.9	116	18%	**13.13%**	0.07	1	196%	60.1	870	7%	**0.12%**	0.1	2	138.59%	0.23%
CZ-27		√		25721	0.2	3	113%	**0.07%**	13.3	199	14%	290.3	4023	3%	**4.38%**	0.0	0	inf	0.00%
CZ-28		√		25547	0.07	1	196%	**0.04%**	72.3	1060	6%	107.1	1554	5%	**40.30%**	0.1	2	138.59%	0.12%
CZ-29	√			24367	29	410	10%	**13.45%**	0.14	2	139%	215.5	2890	4%	**0.06%**	0.0	0	inf	0.00%
CZ-30				23780	0.07	1	196%	**0.07%**	0.14	2	139%	99.8	1351	5%	**0.14%**	0.2	3	113.16%	0.22%

Table S7. FFPE samples detection with dEGFR39 assay-#2 (G719X&20ins)

Num	ARMS		G719X	20Ins

	G719X	E20 ins	Total Number of droplets	Concentration of $G719X_{MU}$ (copies/μl)	Number of positive droplets	Relative uncertainty	Concentration of $G719X_{WT}$ (copies/μl)	Number of positive droplets	Relative uncertainty	G719X%	Concentration of $20ins_{MU}$ (copies/μl)	Number of positive droplets	Relative uncertainty	Concentration of $EGFR_{WT}$ (copies/μl)	Number of positive droplets	Relative uncertainty	20ins%
CZ-01			22894	0.22	3	113%	111	1441	5%	0.20%	0.37	5	88%	92.5	1208	6%	0.40%
CZ-02		√	28139	0.42	7	74%	374.5	5544	3%	0.11%	3.79	47	29%	344.9	5149	3%	1.10%
CZ-03			25862	0.07	1	196%	101.9	1499	5%	0.07%	0.13	2	139%	84.2	1245	6%	0.15%
CZ-04	√		23398	3.87	53	27%	24.2	330	11%	13.79%	0.22	3	113%	14.1	193	14%	1.54%
CZ-05		√	23575	0.14	2	139%	44.1	601	8%	0.32%	0.58	8	69%	39.2	535	8%	1.46%
CZ-06			24726	0.07	1	196%	194.5	2663	4%	0.04%	0.21	3	113%	176.5	2429	4%	0.12%
CZ-07			24162	0.21	3	113%	54.5	760	7%	0.38%	0	0	inf	38.9	544	8%	0.00%
CZ-08			26811	0.19	3	113%	195.7	2904	4%	0.10%	0.57	9	65%	172	2570	4%	0.33%
CZ-09			27706	0.12	2	139%	219.1	3338	3%	0.05%	0.68	11	59%	189.2	2907	4%	0.36%
CZ-10		√	23504	0.22	3	113%	50.9	691	7%	0.43%	0.48	5	87%	40.5	551	8%	1.19%
CZ-11			25895	0.2	3	113%	204.1	2919	4%	0.10%	0.2	3	113%	181.8	2616	4%	0.11%
CZ-12			26794	0.13	2	139%	184.8	2750	4%	0.07%	0.51	8	69%	160.4	2403	4%	0.32%
CZ-13			26107	0.07	1	196%	86.3	1288	5%	0.08%	0.33	5	88%	72.3	1083	6%	0.45%
CZ-14			24803	0.21	3	113%	125.2	1755	5%	0.17%	0.28	4	98%	110.1	1550	5%	0.25%
CZ-15			25605	0.13	2	139%	138.7	1998	4%	0.09%	0	0	inf	121.9	1765	5%	0.00%
CZ-16		√	23248	0.07	1	196%	155.9	2029	4%	0.04%	0.88	12	57%	122.1	1605	5%	0.72%
CZ-17	√		26102	0.44	5	88%	36.1	546	8%	1.22%	0	0	inf	28.5	433	9%	0.00%
CZ-18			26393	0.06	1	196%	216.5	3144	4%	0.03%	0.45	7	74%	193.3	2826	4%	0.23%
CZ-19			27788	0.12	2	139%	126.9	1991	4%	0.09%	0.61	10	62%	115	1810	5%	0.53%
CZ-20			23357	0.07	1	196%	120.7	1595	5%	0.06%	0.29	4	98%	103	1368	5%	0.28%
CZ-21		√	24926	0	0	inf	97.6	1385	5%	0.00%	10.6	154	16%	76.7	1096	6%	12.14%
CZ-22		√	21621	0.08	1	196%	14.3	180	15%	0.56%	0.32	4	98%	9.66	122	18%	3.21%
CZ-23			22183	0.31	4	98%	141.1	1760	5%	0.22%	1	13	54%	125.7	1575	5%	0.79%
CZ-24			21339	0.08	1	196%	92.4	1125	6%	0.09%	0	0	inf	89.0	1084	6%	0.00%
CZ-25			22472	0	0	inf	69.4	896	7%	0.00%	0.08	1	196%	58.2	754	7%	0.13%
CZ-26			22421	0.15	2	139%	98.6	1258	6%	0.15%	0	0	inf	88.7	1136	6%	0.00%
CZ-27			22376	0	0	inf	79.9	1023	6%	0.00%	0.23	3	113%	74.4	954	6%	0.31%
CZ-28			22621	0.08	1	196%	27.8	365	10%	0.27%	0	0	inf	18.7	247	12%	0.00%
CZ-29			23514	0.07	1	196%	62.2	842	7%	0.12%	0.07	1	196%	54.5	739	7%	0.13%
CZ-30			20218	0	0	inf	187.5	2103	4%	0.00%	0.17	2	139%	168.0	1895	5%	0.10%

Table S8. FFPE samples detection with dEGFR39 assay-#3 (T790M/C797S)

Num	ARMS		Total Number of droplets	T790M							$C797S_{TRANS}$			
	T790M	C797S		Concentration of $T790M_{Mu}$ (copies/μl)	Number of positive droplets	Relative uncertainty	Concentration of $T790M_{WT}$ (copies/μl)	Number of positive droplets	Relative uncertainty	$T790M_{Mu}$%	Concentration of $C797S_{TRANS}$ (copies/μl)	Number of positive droplets	Relative uncertainty	$C797S_{TRANS}$%
CZ-01			24644	0.07	1	196%	57.3	814	7%	0.12%	0	0	inf	0.00%
CZ-02			23847	0.79	11	59%	387.9	4848	3%	0.20%	0.07	1	196%	0.02%
CZ-03			23660	0.07	1	196%	59.6	812	7%	0.12%	0.14	2	139%	0.24%
CZ-04			22062	0.08	1	196%	105.2	1319	5%	0.08%	0	0	inf	0.00%
CZ-05			25823	0.07	1	196%	45.8	684	7%	0.15%	0	0	inf	0.00%
CZ-06			20323	0.25	3	113%	248.8	2757	4%	0.10%	0.08	1	196%	0.03%
CZ-07			24783	0	0	inf	122.8	1721	5%	0.00%	0	0	inf	0.00%
CZ-08	√		26234	2.47	38	32%	239.1	3429	3%	1.02%	0.07	1	196%	0.03%
CZ-09			26694	0.45	7	74%	325.6	4636	3%	0.14%	0.06	1	196%	0.02%
CZ-10			19211	0.09	1	196%	105.5	1152	6%	0.09%	0	0	inf	0.00%
CZ-11			23525	0.36	5	88%	275.2	3503	3%	0.13%	0	0	inf	0.00%
CZ-12			22991	0.15	2	139%	102.4	1339	5%	0.15%	0.07	1	196%	0.07%
CZ-13			24778	0	0	inf	49.1	703	7%	0.00%	0	0	inf	0.00%
CZ-14			22722	0.08	1	196%	85	1104	6%	0.09%	0	0	inf	0.00%
CZ-15			23075	0.07	1	196%	192.8	2465	4%	0.04%	0	0	inf	0.00%
CZ-16			23916	0	0	inf	72.1	989	6%	0.00%	0.07	1	196%	0.10%
CZ-17			22895	0.22	3	113%	306.9	3768	3%	0.07%	0	0	inf	0.00%
CZ-18			22725	0.23	3	113%	110.5	1425	5%	0.21%	0	0	inf	0.00%
CZ-19			22417	0	0	inf	65.5	844	7%	0.00%	0.08	1	196%	0.12%
CZ-20			21110	0.08	1	196%	102	1225	6%	0.08%	0.08	1	196%	0.08%
CZ-21			25717	0.13	2	139%	100.2	1466	5%	0.13%	0	0	inf	0.00%
CZ-22			21210	0	0	inf	27.7	341	11%	0.00%	0	0	inf	0.00%
CZ-23			22523	0.08	1	inf	150.2	1898	5%	0.05%	0.08	1	196%	0.05%
CZ-24			22396	0.08	1	inf	105.5	1342	5%	0.07%	0.08	1	196%	0.07%
CZ-25			24373	0	0	inf	68.4	957	6%	0.00%	0	0	inf	0.00%
CZ-26			23622	0.14	2	inf	108.3	1452	5%	0.13%	0.14	2	139%	0.13%
CZ-27			21375	0.08	1	inf	93.1	1135	6%	0.09%	0	0	inf	0.00%
CZ-28			22218	0	0	inf	44.4	571	8%	0.00%	0	0	inf	0.00%
CZ-29			24296	0.14	2	inf	65.7	917	6%	0.21%	0.07	1	196%	0.11%
CZ-30			22122	0.08	1	inf	174.1	2145	4%	0.04%	0	0	inf	0.00%

Table S9. The histopathological characteristics of patients

Feature	n	Tumor EGFR status

		Pos		Neg		P-Value
		n	%	n	%	
**Total**	33	17	51.52%	16	48.48%	
**Age**						
≥63	17	9	27.27%	8	24.24%	0.866
< 63	16	8	24.24%	8	24.24%	
**Gender**						
Male	18	10	30.30%	8	24.24%	0.611
Female	15	7	21.21%	8	24.24%	
**Smoking status**						
Never smoker	21	11	33.33%	10	30.30%	0.895
Smoker	12	6	18.18%	6	18.18%	
**Stage**						
I-II	19	8	24.24%	11	33.33%	
III	4	2	6.06%	2	6.06%	0.352
IV	10	7	21.21%	3	9.09%	
**Depth of tumor invasion**						
T1	8	5	15.15%	3	9.09%	0.473
T2, T3, T4	25	12	36.36%	13	39.39%	
**N stage**						
N0	11	5	15.15%	6	18.18%	0.622
N1, N2	22	12	36.36%	10	30.30%	
**Distant metastasis**						
M0	21	9	27.27%	12	36.36%	0.185
M1	12	8	24.24%	4	12.12%	

Table S10. Plasma samples detection with dEGFR39 assay-#1 (L858R/L861Q/19del).

Num	ARMS			Total Number of droplets	L858R/L861Q				19Del							S768I			
	L858R / L861Q	E19 del	S768I		Concentration of $L858R_{MU}$/ $L861Q_{MU}$ (copies/μl)	Number of positive droplets	Relative uncertainty	Abundance	Concentration of $19Del_{MU}$ (copies/μl)	Number of positive droplets	Relative uncertainty	Concentration of $19Del_{Ref}$ (copies/μl)	Number of positive droplets	Relative uncertainty	Abundance	Concentration of S768I (copies/μl)	Number of positive droplets	Relative uncertainty	Abundance
P-01				31310	0	0	inf	0.00%	0	0	inf	54.4	743	7%	0.00%	0	0	inf	0.00%
P-02	1			28303	1.04	13	54%	25.00%	0	0	inf	4.16	52	27%	0.00%	0	0	inf	0.00%
P-03				23777	0	0	inf	0.00%	0	0	inf	15.7	218	13%	0.00%	0	0	inf	0.00%
P-04		1		21749	0	0	inf	0.00%	0.47	6	80%	27.6	349	10%	1.67%	0	0	inf	0.00%
P-05				27785	0	0	inf	0.00%	0	0	inf	42.7	519	9%	0.00%	0	0	inf	0.00%
P-06		1		28523	0	0	inf	0.00%	0.32	4	98%	13.1	165	15%	2.38%	0	0	inf	0.00%
P-07	1			30731	3	54	27%	13.27%	0	0	inf	22.6	304	11%	0.00%	0	0	inf	0.00%
P-08				27220	0	0	inf	0.00%	0	0	inf	6.24	75	23%	0.00%	0	0	inf	0.00%
P-09				26568	0	0	inf	0.00%	0	0	inf	24.8	289	12%	0.00%	0	0	inf	0.00%
P-10				27077	0	0	inf	0.00%	0	0	inf	13.9	166	15%	0.00%	0	0	inf	0.00%
P-11				26545	0	0	inf	0.00%	0	0	inf	39.8	612	8%	0.00%	0	0	inf	0.00%
P-12		1		31072	0	0	inf	0.00%	1.09	15	51%	18.2	249	12%	5.65%	0	0	inf	0.00%
P-13				23456	0	0	inf	0.00%	0	0	inf	20.4	210	14%	0.00%	0	0	inf	0.00%
P-14				31273	0	0	inf	0.00%	0.22	3	113%	33.9	465	9%	0.64%	0	0	inf	0.00%
P-15	1			30731	3.11	56	26%	13.76%	0	0	inf	22.6	404	10%	0.00%	0	0	inf	0.00%
P-16				27791	0	0	inf	0.00%	0	0	inf	13.8	169	15%	0.00%	0	0	inf	0.00%
P-17		1		25175	0	0	inf	0.00%	4.55	67	24%	45.7	665	8%	9.05%	0	0	inf	0.00%
P-18	1			27775	0.37	6	80%	1.94%	0	0	inf	19.1	309	11%	0.00%	0	0	inf	0.00%
P-19		1		26873	0.19	3	113%	0.74%	0.25	3	113%	33.56	394	10%	0.75%	0	0	inf	0.00%
P-20				28978	0	0	inf	0.00%	0.18	3	113%	23.3	393	10%	0.77%	0	0	inf	0.00%
P-21	1			27559	3.66	59	26%	13.56%	0.19	3	113%	26.8	430	9%	0.70%	0	0	inf	0.00%
P-22				25003	0	0	inf	0.00%	0	0	196%	358	4731	3%	0.00%	0	0	inf	0.00%
P-23	1			27890	9.6	118	18%	55.17%	0	0	inf	17.4	213	13%	0.00%	0	0	inf	0.00%
P-24			1	27181	0.19	3	113%	0.42%	0	0	139%	45.2	711	7%	0.00%	0.33	5	0.877	1.15%
P-25		1		25798	0	0	inf	0.00%	0.61	7	74%	28.5	323	11%	2.10%	0	0	inf	0.00%
P-26				17284	0	0	inf	0.00%	0	0	inf	11.4	115	18%	0.00%	0	0	inf	0.00%
P-27				28080	0.49	6	80%	0.87%	0	0	inf	56.1	687	7%	0.00%	0	0	inf	0.00%
P-28				27324	0	0	inf	0.00%	0	0	inf	28.6	454	9%	0.00%	0	0	inf	0.00%
P-29				16616	0	0	inf	0.00%	0	0	inf	57.7	552	8%	0.00%	0	0	inf	0.00%
P-30				17040	0	0	196%	0.00%	0	0	196%	24	238	13%	0.00%	0	0	inf	0.00%
P-31				25731	0	0	inf	0.00%	0	0	inf	36.4	543	8%	0.00%	0	0	inf	0.00%

**P-32**		**1**		**23861**	**0**	**0**	**inf**	**0.00%**	**0.57**	**8**	**69%**	**30.8**	**427**	**9%**	**1.82%**	**0**	**0**	**inf**	**0.00%**
**P-33**		**1**		**22135**	**0**	**0**	**inf**	**0.00%**	**6.64**	**86**	**21%**	**117.2**	**1469**	**5%**	**5.36%**	**0**	**0**	**inf**	**0.00%**

Table S11. Plasma samples detection with dEGFR39 assay-#2 (G719X&20ins)

**Num**	**ARMS**		**Total Number of droplets**	**G719X**							**20Ins**						
	**G719X**	**E20 ins**		**Concentration of $G719X_{MU}$ (copies/µl)**	**Number of positive droplets**	**Relative uncertainty**	**Concentration of $G719X_{WT}$ (copies/µl)**	**Number of positive droplets**	**Relative uncertainty**	**G719X%**	**Concentration of $20ins_{MU}$ (copies/µl)**	**Number of positive droplets**	**Relative uncertainty**	**Concentration of $20Ins_{Ref}$ (copies/µl)**	**Number of positive droplets**	**Relative uncertainty**	**20ins%**
P-01			25415	0	0	inf	36.72	541	8%	0.00%	0	0	inf	38.23	563	8%	0.00%
P-02			25413	0	0	inf	37.14	547	8%	0.00%	0	0	inf	38.65	569	8%	0.00%
P-03			22228	0	0	inf	64	818	7%	0.00%	0	0	inf	59.22	758	7%	0.00%
P-04			29824	0	0	inf	32.4	424	10%	0.00%	0	0	inf	30.84	534	8%	0.00%
P-05			26870	0	0	inf	33.3	393	10%	0.00%	0	0	inf	25.98	406	10%	0.00%
P-06			29149	0	0	inf	8.01	103	19%	0.00%	0	0	inf	5.57	95	20%	0.00%
P-07		1	22704	0	0	inf	18.21	241	13%	0.00%	1.3	13	54%	18.5	185	14%	6.57%
P-08			29324	0	0	inf	6.57	85	21%	0.00%	0	0	inf	4.72	81	22%	0.00%
P-09			28978	0	0	inf	23.5	396	10%	0.00%	0	0	inf	21.57	364	10%	0.00%
P-10			31046	0	0	inf	10.2	139	17%	0.00%	0	0	inf	6.66	121	18%	0.00%
P-11			23856	0	0	inf	28.1	294	11%	0.00%	0	0	inf	21.96	305	11%	0.00%
P-12			23744	0	0	inf	33.3	459	9%	0.00%	0	0	inf	27.54	380	10%	0.00%
P-13			29162	0	0	inf	24.8	318	11%	0.00%	0	0	inf	17.94	305	11%	0.00%
P-14			28386	0	0	inf	28.7	358	10%	0.00%	0	0	inf	18.8	311	11%	0.00%
P-15			26942	0	0	inf	36.9	576	8%	0.00%	0	0	inf	32.68	511	9%	0.00%
P-16			22541	0	0	inf	96.97	1245	6%	0.00%	0.076	1	196%	105.24	1348	5%	0.07%
P-17			27303	0	0	inf	6.74	78	22%	0.00%	0	0	inf	5.38	86	21%	0.00%
P-18			25417	0	0	inf	23.94	354	10%	0.00%	0	0	inf	23.12	342	11%	0.00%
P-19			27441	0	0	inf	26	313	11%	0.00%	0	0	inf	20.84	333	11%	0.00%
P-20			28627	0	0	inf	11.1	140	17%	0.00%	0	0	inf	6.33	106	19%	0.00%
P-21			27579	0.06	1	196%	44.6	712	7%	0.13%	0	0	inf	54.32	864	7%	0.00%
P-22			26548	0	0	inf	87.11	1321	5%	0.00%	0	0	inf	95.25	1441	5%	0.00%
P-23			33113	0	0	inf	57.1	825	7%	0.00%	0	0	inf	33.94	652	8%	0.00%
P-24			25801	0	0	inf	14	211	13%	0.00%	0	0	inf	53.48	796	7%	0.00%
P-25			26010	0	0	inf	48.1	547	8%	0.00%	0	0	inf	43.93	661	8%	0.00%
P-26			23226	0	0	inf	24.3	328	11%	0.00%	0	0	inf	24.13	326	11%	0.00%
P-27			25560	0	0	inf	69.6	1022	6%	0.00%	0	0	inf	67.98	998	6%	0.00%
P-28			28071	0	0	inf	39.5	643	8%	0.00%	0	0	inf	40.17	653	8%	0.00%
P-29			28812	0	0	inf	76.9	962	6%	0.00%	0	0	inf	60.78	1008	6%	0.00%

P-30			29198	0	0	inf	108.2	1363	5%	0.00%	0.058	1	196%	85.4	1425	5%	0.07%
P-31			19486	0	0	inf	4.3	49	28%	0.00%	0	0	inf	16.9	192	14%	0.00%
P-32			27303	0	0	inf	6.74	78	22%	0.00%	0	0	inf	5.45	87	21%	0.00%
P-33			22580	0	0	inf	6.51	86	21%	0.00%	0	0	inf	7.12	94	20%	0.00%

Table S12. Plasma samples detection with dEGFR39 assay-#3 (T790M/C797S)

Num	ARMS		Total Number of droplets	T790M							$C797S_{TRANS}$			
	T790M	C797S		Concentration of $T790M_{Mu}$ (copies/μl)	Number of positive droplets	Relative uncertainty	Concentration of $T790M_{WT}$ (copies/μl)	Number of positive droplets	Relative uncertainty	$T790M_{Mu}$%	Concentration of $C797S_{TRANS}$ (copies/μl)	Number of positive droplets	Relative uncertainty	$C797S_{TRANS}$%
P-01			24722	0	0	inf	43.3	468	9%	0.00%	0	0	inf	0.00%
P-02			28587	0	0	inf	29.3	487	9%	0.00%	0	0	inf	0.00%
P-03			26434	0.34	4	98%	63.4	964	6%	0.54%	0.06	1	196%	0.10%
P-04			28071	0	0	inf	34.8	566	8%	0.00%	0	0	inf	0.00%
P-05			21203	0	0	inf	29.6	365	10%	0.00%	0	0	inf	0.00%
P-06	1		25127	0.63	7	74%	7.67	85	21%	7.59%	0	0	inf	0.00%
P-07			30200	0	0	inf	12.4	165	15%	0.00%	0	0	inf	0.00%
P-08	1		25396	0.36	4	98%	15.6	174	16%	2.31%	0	0	inf	0.00%
P-09			31266	0	0	inf	19.4	267	12%	0.00%	0	0	inf	0.00%
P-10			28558	0.12	2	139%	9.77	123	18%	1.21%	0	0	inf	0.00%
P-11			17621	0	0	inf	26.7	274	12%	0.00%	0	0	inf	0.00%
P-12			30075	0	0	inf	25.9	342	11%	0.00%	0	0	inf	0.00%
P-13			19566	0	0	inf	20.2	174	15%	0.00%	0	0	inf	0.00%
P-14			30204	0.06	1	196%	22.4	298	11%	0.25%	0	0	inf	0.00%
P-15			27117	0	0	inf	34.8	548	8%	0.00%	0	0	inf	0.00%
P-16			25530	0	0	inf	77.8	1137	6%	0.00%	0.07	1	196%	0.09%
P-17			16352	0	0	inf	5.4	39	31%	0.00%	0	0	inf	0.00%
P-18			21254	0.32	3	113%	29.9	279	12%	1.07%	0	0	inf	0.00%
P-19			26873	0	0	inf	25.6	400	10%	0.00%	0	0	inf	0.00%
P-20			27494	0.06	1	196%	7.75	94	20%	0.79%	0	0	inf	0.00%
P-21			27662	0	0	inf	56	893	7%	0.00%	0	0	inf	0.00%
P-22			25573	0.44	5	88%	78	1142	6%	0.56%	0	0	inf	0.00%
P-23			22228	0	0	inf	53.2	682	8%	0.00%	0	0	inf	0.00%
P-24			26008	0.07	1	196%	28.7	433	9%	0.23%	0	0	inf	0.00%
P-25			27579	0	0	inf	42.4	676	8%	0.00%	0	0	inf	0.00%
P-26			17924	0	0	inf	18	188	14%	0.00%	0	0	inf	0.00%
P-27			28080	0	0	inf	56.1	687	7%	0.00%	0	0	inf	0.00%
P-28			27091	0	0	inf	36.5	573	8%	0.00%	0	0	inf	0.00%

P-29	1		24267	1.59	17	48%	148.8	2026	4%	1.07%	0	0	inf	0.00%
P-30			21305	0	0	inf	137.9	1654	5%	0.00%	0	0	inf	0.00%
P-31			23719	0	0	inf	4.9	68	24%	0.00%	0	0	inf	0.00%
P-32			18922	0	0	inf	6.87	76	23%	0.00%	0	0	inf	0.00%
P-33			28187	0	0	inf	5.14	64	25%	0.00%	0	0	inf	0.00%

Table S13. Dynamic monitor with dEGFR39 panel-#1 (L858R/L861Q/19del)

Num	Date	ARMS			Total Number of droplets	L858R/L861Q				19Del							S768I			
		L858R / L861Q	E19 del	S768I		Concentration of $L858R_{MU}$/ $L861Q_{MU}$ (copies/μl)	Number of positive droplets	Relative uncertainty	Abundance	Concentration of $19Del_{MU}$ (copies/μl)	Number of positive droplets	Relative uncertainty	Concentration of $19Del_{Ref}$ (copies/μl)	Number of positive droplets	Relative uncertainty	Abundance	Concentration of S768I (copies/μl)	Number of positive droplets	Relative uncertainty	Abundance
P-12	0		1		31072	0	0	inf	0.00%	1.09	15	51%	18.2	249	12%	5.65%	0	0	inf	0.00%
	314				29828	0	0	inf	0.00%	0	0	inf	38.9	508	9%	0.00%	0	0	inf	0.00%
	444				16271	0	0	inf	0.00%	37.1	261	12%	64.9	460	9%	36.37%	0	0	inf	0.00%
	539				29003	0	0	inf	0.00%	24.7	315	11%	65.4	826	7%	27.41%	0	0	inf	0.00%
P-15	0	1			30731	3.11	56	26%	13.76%	0	0	inf	22.6	404	10%	0.00%	0	0	inf	0.00%
	368				29882	0.15	2	139%	1.62%	0	0	inf	9.26	122	139%	0.00%	0	0	inf	0.00%
	432				30529	0.67	9	65%	3.45%	0	0	inf	19.4	261	65%	0.00%	0	0	inf	0.00%
	489				24687	4.96	54	2666%	13.97%	0	0	inf	35.5	384	27%	0.00%	0	0	inf	0.00%
	552				25003	15.05	165	14%	42.02%	0	0	inf	35.7	472	14%	0.00%	0	0	inf	0.00%
P-23	0	1			27890	2.61	31	35%	14.94%	0	0	inf	17.4	213	13%	0.00%	0	0	inf	0.00%
	187				30643	0.66	9	65%	4.34%	0	0	inf	15.2	205	14%	0.00%	0	0	inf	0.00%
	300	1			27749	0.65	8	69%	4.39%	0	0	inf	14.8	181	15%	0.00%	0	0	inf	0.00%
	412				26885	2.16	34	34%	2.66%	0	0	inf	81.2	1249	6%	0.00%	0	0	inf	0.00%
P-04	0		1		21749	0	0	inf	0.00%	0.47	6	80%	27.6	349	10%	1.67%	0	0	inf	0.00%
	95				29346	0	0	inf	0.00%	0.54	7	74%	11.4	148	16%	4.52%	0	0	inf	0.00%
	133				26827	0	0	inf	0.00%	1.7	19	45%	18.6	219	13%	8.37%	0	0	inf	0.00%
	387				26942	0	0	inf	0.00%	3.55	56	26%	36.9	576	8%	8.78%	0	0	inf	0.00%
P-25	0		1		25798	0	0	inf	0.00%	0.61	7	74%	28.5	323	11%	2.10%	0	0	inf	0.00%
	194				31849	0	0	inf	0.00%	0	0	inf	39.1	546	8%	0.00%	0	0	inf	0.00%
	251				30274	0	0	inf	0.00%	1	13	54%	17.2	229	13%	5.49%	0	0	inf	0.00%
	294				27174	0	0	inf	0.00%	15.2	176	15%	94.4	1110	6%	13.87%	0	0	inf	0.00%
P-07	0	1			30731	3.03	41	31%	13.27%	0	0	inf	22.6	404	10%	0.00%	0	0	inf	0.00%
	452				24078	1.13	12	57%	5.31%	0	0	inf	21.3	226	13%	0.00%	0	0	inf	0.00%
	581				32345	0.35	5	88%	6.67%	0	0	inf	5.25	75	23%	0.00%	0	0	inf	0.00%
	666				27775	0.37	6	80%	1.94%	0	0	inf	19.1	309	11%	0.00%	0	0	inf	0.00%

Table S14. Dynamic monitor with dEGFR39 panel-#2 (G719X&20ins)

Num	Day	ARMS		Total Number of droplets	G719X							20Ins						
		G719X	E20 ins		Concentration of $G719X_{MU}$ (copies/µl)	Number of positive droplets	Relative uncertainty	Concentration of $G719X_{WT}$ (copies/µl)	Number of positive droplets	Relative uncertainty	G719X%	Concentration of $20ins_{MU}$ (copies/µl)	Number of positive droplets	Relative uncertainty	Concentration of $20Ins_{Ref}$ (copies/µl)	Number of positive droplets	Relative uncertainty	20ins%
P-12	0			23744	0	0	inf	33.30	459	9%	0.00%	0	0	inf	27.54	380	10%	0.00%
	314			24516	0	0	inf	52.67	745	7%	0.00%	0	0	inf	46.15	654	8%	0.00%
	444			25648	0	0	inf	40.74	605	8%	0.00%	0	0	inf	37.75	561	8%	0.00%
	539			23698	0	0	inf	30.01	413	10%	0.00%	0	0	inf	25.69	354	10%	0.00%
P-15	0			26942	0	0	inf	36.90	576	8%	0.00%	0	0	inf	32.68	511	9%	0.00%
	368			21456	0	0	inf	41.55	516	9%	0.00%	0	0	inf	47.51	589	8%	0.00%
	432			21546	0	0	inf	27.63	346	11%	0.00%	0	0	inf	26.18	328	11%	0.00%
	489			25874	0	0	inf	26.66	401	10%	0.00%	0	0	inf	27.60	415	10%	0.00%
	552			21368	0	0	inf	33.80	419	10%	0.00%	0	0	inf	29.32	364	10%	0.00%
P-23	0			33113	0	0	inf	57.10	825	7%	0.00%	0	0	inf	33.94	652	8%	0.00%
	187			25746	0	0	inf	27.06	405	10%	0.00%	0	0	inf	30.57	457	9%	0.00%
	300			23654	0	0	inf	29.55	406	10%	0.00%	0	0	inf	26.54	365	10%	0.00%
	412			25648	0	0	inf	20.15	301	11%	0.00%	0	0	inf	14.30	214	13%	0.00%
P-04	0			29824	0	0	inf	32.40	424	10%	0.00%	0	0	inf	30.84	534	8%	0.00%
	95			22214	0	0	inf	19.01	246	12%	0.00%	0	0	inf	12.96	168	15%	0.00%
	133			25541	0	0	inf	20.57	306	11%	0.00%	0	0	inf	16.59	247	12%	0.00%
	387			23684	0	0	inf	51.06	698	7%	0.00%	0	0	inf	54.10	739	7%	0.00%
P-25	0			26010	0	0	inf	48.10	547	8%	0.00%	0	0	inf	43.93	661	8%	0.00%
	194			22214	0	0	inf	24.56	365	10%	0.00%	0	0	inf	12.96	168	15%	0.00%
	251			25541	0	0	inf	21.18	301	11%	0.00%	0	0	inf	16.59	247	12%	0.00%
	294			23684	0	0	inf	22.58	334	11%	0.00%	0	0	inf	54.10	739	7%	0.00%
P-07	0			21854	0	0	inf	17.74	226	13%	0.00%	0	0	inf	9.71	124	18%	0.00%
	452		1	22704	0	0	inf	18.21	241	13%	0.00%	1.3	13	54%	18.50	185	14%	6.57%
	581			22365	0	0	inf	17.26	225	13%	0.00%	0	0	inf	14.48	189	14%	0.00%
	666			25421	0	0	inf	21.89	324	11%	0.00%	0	0	inf	17.14	254	12%	0.00%

Table S15. Dynamic monitor with dEGFR39 panel-#3 (T790M/ C797S)

Num	Day	ARMS		Total Number of droplets	T790M							$C797S_{TRANS}$			
		T790M	C797S		Concentration of $T790M_{Mu}$ (copies/µl)	Number of positive droplets	Relative uncertainty	Concentration of $T790M_{WT}$ (copies/µl)	Number of positive droplets	Relative uncertainty	$T790M_{Mu}$%	Concentration of $C797S_{TRANS}$ (copies/µl)	Number of positive droplets	Relative uncertainty	$C797S_{TRANS}$%
P-12	0			30075	0	0	inf	25.9	342	11%	0.00%	0	0	inf	0.00%
	314			27657	0	0	inf	30.4	369	10%	0.00%	0	0	inf	0.00%

	444			32640	0	0	inf	43	615	8%	0.00%	0	0	inf	0.00%
	539			23933	0	0	inf	47.1	493	9%	0.00%	0	0	inf	0.00%
P-15	0			27117	0	0	inf	34.8	548	8%	0.00%	0	0	inf	0.00%
	368			30292	0	0	inf	26.8	356	10%	0.00%	0	0	inf	0.00%
	432			31247	0.07	1	196%	11.3	181	15%	0.62%	0	0	inf	0.00%
	489	1		19615	1.31	15	51%	22.6	258	12%	5.48%	0	0	inf	0.00%
	552	1		23450	15.4	203	14%	28.8	365	10%	34.84%	0	0	inf	0.00%
P-23	0			22228	0	0	inf	53.2	682	8%	0.00%	0	0	inf	0.00%
	187			27425	0.33	4	98%	13	157	16%	2.48%	0	0	inf	0.00%
	300	1		29934	1.97	26	38%	46.7	611	8%	4.05%	0	0	inf	0.00%
	412	1		25648	2.06	31	35%	46.5	690	7%	4.24%	0	0	inf	0.00%
P-04	0			28071	0	0	inf	34.8	566	8%	0.00%	0	0	inf	0.00%
	95			30732	0	0	inf	22.1	299	11%	0.00%	0	0	inf	0.00%
	133			28239	0	0	inf	13.3	166	15%	0.00%	0	0	inf	0.00%
	387			22687	0	0	inf	47.8	627	8%	0.00%	0	0	inf	0.00%
P-25	0			27579	0	0	inf	42.4	676	8%	0.00%	0	0	inf	0.00%
	194			20294	0	0	inf	40.7	362	10%	0.00%	0	0	inf	0.00%
	251	1		30422	0.3	4	98%	12.5	168	15%	2.34%	0	0	inf	0.00%
	294	1		18960	2.97	33	34%	39	428	9%	7.08%	0	0	inf	0.00%
P-07	0			30200	0	0	inf	12.4	165	15%	0.00%	0	0	inf	0.00%
	452			29146	0	0	inf	11.8	152	16%	0.00%	0	0	inf	0.00%
	581			28080	0	0	inf	4.84	60	25%	0.00%	0	0	inf	0.00%
	666			29154	0	0	inf	4.82	62	25%	0.00%	0	0	inf	0.00%
